# Polygenic analysis of the effect of common and low-frequency genetic variants on serum uric acid levels in Korean individuals

**DOI:** 10.1038/s41598-020-66064-z

**Published:** 2020-06-08

**Authors:** Sung Kweon Cho, Beomsu Kim, Woojae Myung, Yoosoo Chang, Seungho Ryu, Han-Na Kim, Hyung-Lae Kim, Po-Hsiu Kuo, Cheryl A. Winkler, Hong-Hee Won

**Affiliations:** 1 Samsung Advanced Institute for Health Sciences and Technology (SAIHST), Sungkyunkwan University, Samsung Medical Center, Seoul, Republic of Korea; 2 Molecular Genetic Epidemiology Section, Basic Research Program, Frederick National Laboratory for Cancer Research, Frederick, MD USA; 3 Department of Neuropsychiatry, Seoul National University Bundang Hospital, Seongnam-si, Republic of Korea; 4 Center for Cohort Studies, Total Healthcare Center, Kangbuk Samsung Hospital, Sungkyunkwan University School of Medicine, Seoul, Republic of Korea; 5 Medical Research Institute, Kangbuk Samsung Hospital, Sungkyunkwan University School of Medicine, Seoul, Republic of Korea; 6 Department of Biochemistry, Ewha Womans University, Seoul, Republic of Korea; 7 Department of Public Health & Institute of Epidemiology and Preventive Medicine, College of Public Health, National Taiwan University, Taipei, Taiwan

**Keywords:** Genome-wide association studies, Quantitative trait

## Abstract

Increased serum uric acid (SUA) levels cause gout and are associated with multiple diseases, including chronic kidney disease. Previous genome-wide association studies (GWAS) have identified more than 180 loci that contribute to SUA levels. Here, we investigated genetic determinants of SUA level in the Korean population. We conducted a GWAS for SUA in 6,881 Korean individuals, calculated polygenic risk scores (PRSs) for common variants, and validated the association of low-frequency variants and PRS with SUA levels in 3,194 individuals. We identified two low-frequency and six common independent variants associated with SUA. Despite the overall similar effect sizes of variants in Korean and European populations, the proportion of variance for SUA levels explained by the variants was greater in the Korean population. A rare, nonsense variant *SLC22A12* p.W258X showed the most significant association with reduced SUA levels, and PRSs of common variants associated with SUA levels were significant in multiple Korean cohorts. Interestingly, an East Asian-specific missense variant (rs671) in *ALDH2* displayed a significant association on chromosome 12 with the SUA level. Further genetic epidemiological studies on SUA are needed in ethnically diverse cohorts to investigate rare or low-frequency variants and determine the influence of genetic and environmental factors on SUA.

## Introduction

Uric acid is the final product of purine metabolism in humans^1^. Uric acid is produced primarily in the liver, and 70% of daily uric acid excretion occurs via the kidney^1^. The uric acid transport system in the kidney plays an important role in its excretion^2^, since only 10% of the initially filtered uric acid is excreted through urine^3^. The serum uric acid (SUA) level is determined by the balance of renal clearance, extrarenal clearance in the gut, and purine metabolism in the liver. The absence of the enzyme uricase and the presence of the renal uptake system contribute to higher SUA levels in humans, compared to that in other mammals^1,4^. SUA levels in the general population follow a normal distribution^2^. The physiological normal range of uric acid is 2.5-7.5 and 2.0-6.5 mg/dL for men and women, respectively^5^. The female hormones that influence the renal uptake of uric acid^6,7^ and the effects of genetic variations in *SLC2A9* and *ABCG2*^8,9^ are reported as sex-related factors.

Hyperuricemia is the result of overactive hepatic metabolism and high cell turnover or renal under-excretion/extra-renal under-excretion or a combination of both^10^. Generally, renal and gut under-excretion account for two-thirds^11^ and one-third^12^ of hyperuricemia caused by under-excretion. In addition to environmental factors, genetic factors identified by previous genome-wide association studies (GWASs) have been implicated in the critical process of uric acid excretion and its role in hyperuricemia^13–18^. *SLC2A9* and *ABCG2* were found to be the most prominent loci in a GWAS of more than 143,160 participants of European ancestry^9^ and a trans-ancestry GWAS of 457,690 individuals^19^.

A rare, loss-of-function variant (p.W258X) of *SLC22A12* has been reported to be a founder mutation of familial renal hypouricemia (RHUC1, OMIM #220150) in the Japanese population^20^. An earlier GWAS reported an association between common variants of the *SLC22A12* locus and SUA levels^15^. The loss-of-function variant (p.W258X) could not be identified in the Asian Genetic Epidemiology Network (AGEN) GWAS owing to its rare occurrence in East Asian populations^21^. Recently, rare functional variants (p.R325W, p.R405C, and p.T467M) of the *SLC22A12* gene have been detected in European and African-American populations, highlighting the importance of population-specific rare and common variants^22^.

Global research in GWAS suffers from a Eurocentric bias and, as a consequence, disproportionately underrepresents non-European populations^23,24^. Although trans-ancestry GWASs have identified more than 180 loci associated with SUA^25,26^, the current challenges include unearthing explanations for the remaining missing heritability of SUA by identifying additional genetic variants in diverse populations and a systematic evaluation of differential effects of common and rare variants. Recently, it has been shown that common and low-frequency variants can accurately be imputed (R^2^ ≥ 0.8) using a large imputation panel of the Haplotype Reference Consortium (HRC) consisting of 64,976 haplotypes^27^. Here, we performed a genome-wide association analysis of SUA levels in the Korean population and evaluated the genetic effects on SUA levels using imputed single-nucleotide polymorphism (SNP) chip data. We also calculated polygenic risk scores (PRS) to evaluate the variability of SUA in multiple Korean cohorts.

## Results

### Characteristics of the participants

In total, 6,881 individuals from urban and rural cohorts were included in the discovery phase, and a total of 3,194 individuals from the Ansan-Ansung and Kangbuk Samsung Hospital (KBSMC) cohorts were used for model validation (Table 1). The mean characteristics of the participants were slightly or substantially different between the cohorts. Overall, the urban and Ansan-Ansung cohorts included more males and more participants with hypertension, hyperlipidaemia, and diabetes than the rural cohort, which were included as covariates in the association analysis. Our discovery (*n* = 6,881) and replication (*n* = 3,194) sets consisted of individuals with preserved renal function (eGFR > 60 ml/min); however, we did not exclude participants with hypertension or diabetes.

**Table 1:** Clinical characteristics of the study cohort.

	Urban (*n* = 3,585)	Rural (*n* = 3,296)	Ansan-Ansung (*n* = 1,167)	KBSMC (*n* = 2,027)
Age (years)	53.16 ± 8.35	59.69 ± 10.02	64.61 ± 8.26	39.35 ± 8.87
Gender (male)	1,599 (44.60%)	1,238 (37.56%)	469 (40.19%)	1,138 (56.14%)
BMI (kg/m^2^)	23.97 ± 2.89	23.82 ± 3.05	24.38 ± 3.31	23.18 ± 3.16
Uric acid (mg/dL)	4.82 ± 1.34	4.75 ± 1.35	4.89 ± 1.41	5.30 ± 1.45
eGFR (ml/min)	89.20 ± 13.36	76.52 ± 10.95	68.30 ± 12.49	96.92 ± 16.10
Hypertension	666 (18.58%)	43 (1.30%)	51 (4.37%)	203 (10.01%)
Hyperlipidaemia	222 (6.33%)	4 (0.12%)	33 (2.78%)	—
Diabetes	244 (6.81%)	20 (0.61%)	177 (15.17%)	54 (2.66%)
**Blood pressure (mmHg)**				
Systolic	121.66 ± 14.31	115.82 ± 11.51	127.45 ± 16.46	107.38 ± 12.84
Diastolic	77.03 ± 9.76	75.11 ± 7.61	78.46 ± 9.02	69.19 ± 9.59
**Alcohol intake**				
Former drinkers	172 (4.80%)	182 (5.52%)	95 (8.14%)	—
Current drinkers	1,711 (47.73%)	1,477 (44.81%)	420 (35.99%)	—
**Smoking status**				
Former smokers	651 (18.16%)	505 (15.32%)	201 (17.22%)	392 (19.34%)
Current smokers	525 (14.64%)	553 (16.78%)	151 (12.94%)	274 (13.52%)
Total cholesterol (mg/dL)	197.40 ± 34.97	195.52 ± 35.40	188.06 ± 33.02	192.80 ± 33.47
Triglycerides (mg/dL)	122.81 ± 89.46	137.35 ± 85.95	141.83 ± 99.29	108.48 ± 73.69
HDL cholesterol (mg/dL)	54.56 ± 13.23	45.27 ± 10.87	45.17 ± 11.23	59.59 ± 15.13
FBS (mg/dL)	94.26 ± 24.84	94.11 ± 9.64	99.18 ± 23.76	—

Values are mean ± standard deviation (SD) for continuous data and count (%) for discrete data.

Abbreviations: BMI, body mass index; eGFR, estimated glomerular filtration rate; SBP, systolic blood pressure; DBP, diastolic blood pressure; HDL, high-density lipoprotein; LDL, low-density lipoprotein; FBS, fasting blood sugar.

### Meta-analysis of SUA levels

**Figure 1:**
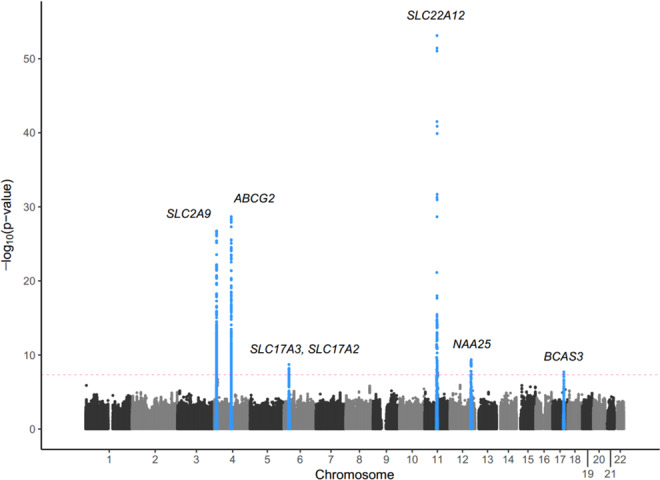
Manhattan plot depicting genome-wide association analysis of serum uric acid. Each dot represents a variant plotted as −log_10_(*P*-value) on the *y* axis against the corresponding variant position on the *x* axis. Dots in blue indicate variants in loci characterised by a genome-wide significance level (shown by the pink dashed line).

We analysed a total of 6,129,701 SNPs in the discovery phase to identify the association with SUA levels in individuals using linear regression and discovered eight independent SNPs (two low-frequency and six common) that reached the threshold of statistical significance (Table 2). In the discovery phase, the inflation factor for the meta-analysis of SUA was 0.998 after performing a genomic control (1.02 without genomic control) (Supplementary Fig. S1). A Manhattan plot of the meta-analysis on SUA levels is shown in Fig. 1. A total of 1,149 SNPs that reached a level of genome-wide significance (*P*-value <5.0 × 10^−8^) for association with SUA levels are listed in Supplementary Table S1, and the 10 independent nonsynonymous SNPs that passed Bonferroni’s correction for 11,600 nonsynonymous SNPs (*P*-value <4.31 × 10^−6^) are listed in Supplementary Table S3. The regional plots for genome-wide significant association are shown in Supplementary Fig. S3. Conditional analyses identified multiple variants, including rs184521656, a low-frequency (minor allele frequency (MAF) = 0.01) intronic variant in *FRMD8*, that reached a level of genome-wide significance for association with SUA levels after conditioning with pre-selected lead SNPs in the flanking region (Supplementary Fig. S4a). Other regions did not show multiple independent signals at the level of genome-wide significance (Supplementary Fig. S4). The results of the main meta-analysis were similar to the simple model that used only sex, age, and the first 10 principal components of genetic variants as covariates, except for the chromosome 17 locus near the *BCAS3* gene, which is a previously reported locus (Supplementary Fig. S5)^9^.

**Table 2:** Lead variants associated with SUA from meta-analysis of GWASs.

SNP	Chr	BP	Nearest gene	Function	A1	A2	β	SE	*P*-value	EAF in each cohort			
										Urban	Rural	Ansan-Ansung	KBSMC
rs121907892	11	64361219	*SLC22A12*	nonsense	A	G	−1.151	0.075	7.43 × 10^−54^	0.009	0.018	0.008	—
rs2231142	4	89052323	*ABCG2*	missense	T	G	0.221	0.020	2.06 × 10^−29^	0.273	0.258	0.266	0.255
rs4529048	4	9997112	*SLC2A9*	intronic	A	C	0.192	0.018	1.81 × 10^−27^	0.579	0.574	0.576	0.564
rs1212146	11	64412877	*NRXN2*	intronic	A	G	−0.130	0.018	9.23 × 10^−14^	0.570	0.567	0.587	0.573
rs116873087 (rs671)	12	112511913	*NAA25* (*ALDH2*, *ATXN2*, *CUX*)	intronic	C	G	−0.150	0.024	4.33 × 10^−10^	0.153	0.167	0.167	0.155
rs566530	6	25878361	*SLC17A3*, *SLC17A2*	intergenic	T	C	−0.145	0.024	1.97 × 10^−09^	0.145	0.160	0.153	0.160
rs9895661	17	59456589	*BCAS3*	intronic	T	C	0.099	0.018	1.96 × 10^−08^	0.519	0.523	—	0.542
rs16890979	4	9922167	*SLC2A9*	missense	T	C	−0.473	0.095	5.86 × 10^−07^	0.008	0.009	0.011	—

Previously reported genes near rs116873087 include ALDH2, ATXN2, and CUX. rs671 was found to be in high linkage disequilibrium with rs116873087 (r2 = 0.985).

Abbreviations: Chr, chromosome number; BP, base position; A1, effective allele; A2, non-effective allele; EAF, effective allele frequency; β, coefficient of each SNP obtained by linear regression; SE, standard error.

Of note, a rare nonsense variant in the *SLC22A12* locus showed the strongest association with SUA levels (rs121907892, *P*-value = 7.4 × 10^−54^; β = −1.15 mg/dl; standard error (SE) = 0.07 mg/dl) in the discovery phase (Supplementary Fig. S2). The locus was monomorphic in most populations, but the variant was found to be present at a very low frequency in only East Asian populations. This variant has been reported in a previous exome-wide association study in the Japanese population^28^. It is also present in very low linkage disequilibrium with most of the other neighbouring variants. Another low-frequency missense variant in *SLC2A9*, previously identified in GWAS studies^9,29^, also showed significant associations with SUA levels (rs16890979, *P*-value = 5.86 × 10^−7^; β = −0.47 mg/dl; SE = 0.09 mg/dl) in the discovery phase. This variant is a common polymorphism observed in most tested populations. Other significant common variants were found to be located near the *ABCG2*, *SLC2A9*, *NRXN2*, *NAA25*, *SLC17A3*-*SLC17A2*, and *BCAS3* genes (Supplementary Table S5). Although most of these loci were previously reported in GWASs, the lead SNPs identified in the present study are different from those reported in European populations (Supplementary Fig. S3)^9^.

### Sex-stratified meta-analysis of SUA levels

In addition to the lead variants in *ABCG2* (rs2231142, male; *P*-value = 5.9 × 10^−14^; β = 0.27 mg/dl; SE = 0.04 mg/dl, female; *P*-value = 4.3 × 10^−18^; β = 0.19 mg/dl; SE = 0.02 mg/dl) and *SLC2A9* (rs4529048, male; *P*-value = 6.2 × 10^−06^; β = 0.15 mg/dl; SE = 0.03 mg/dl, female; *P*-value = 2.2 × 10^−31^; β = 0.23 mg/dl; SE = 0.02 mg/dl) that were previously known to show sex-specific patterns^8,9^, our sex-stratified analysis identified intronic variants in *CDH13* on chromosome 16 that were significant in the analysis of female participants (rs8063966, *P*-value = 1.6 × 10^−08^; β = −0.18 mg/dl; SE = 0.03 mg/dl) (Supplementary Fig. S7).

### Alcohol intake-adjusted associations in the region on chromosome 12

We performed meta-analysis of SUA levels using the alcohol intake-adjusted association results to check for the influence of the identified loci on uric acid levels via alcohol-related pathways. The variants in the chromosome 12 region did not show significant associations after adjusting alcohol intake, whereas other loci remained significant after this adjustment (Supplementary Fig. S6a,b). In particular, the chromosome 12 region was significant in the genome-wide meta-analysis of alcohol intake (Supplementary Fig. S6c). A common, missense variant in *ALDH2* in this region, known to be associated with alcohol metabolism and drinking behaviour^30,31^, showed the strongest association with alcohol intake (rs671, *P*-value = 9.4 × 10^−101^; odds ratio = 0.049; 95% confidence interval = 0.037 to 0.065) and was in high linkage disequilibrium with rs116873087, the lead SNP in *NAA25* (*r*^2^ = 0.985). Variants in the *ATXN2* and *CUX2* genes associated with SUA levels also showed association with alcohol intake (Supplementary Fig. S6d).

### Comparison of significant variants identified in our study and the European study

Allele frequencies, haplotype structure, and effect sizes of the SNPs identified in the Korean cohorts may be different from those reported in European populations, which may lead to differences in the proportion of variance explained by the variants identified in the Korean and European populations. We compared the effect sizes and the proportion of variance for phenotype explained by a given SNP (PVE) for the significant variant sets from our study and from the Global Urate Genetics Consortium (GUGC) for the European population. The effect sizes of the lead SNPs in our study were approximately linear to those in the GUGC study and showed the same direction of effects as the GUGC study. Of the 27 significant lead variants from the GUGC study, 22 showed significant associations (*P*-value <0.05) in our study and the same direction of effects between the two studies.

**Figure 2:**
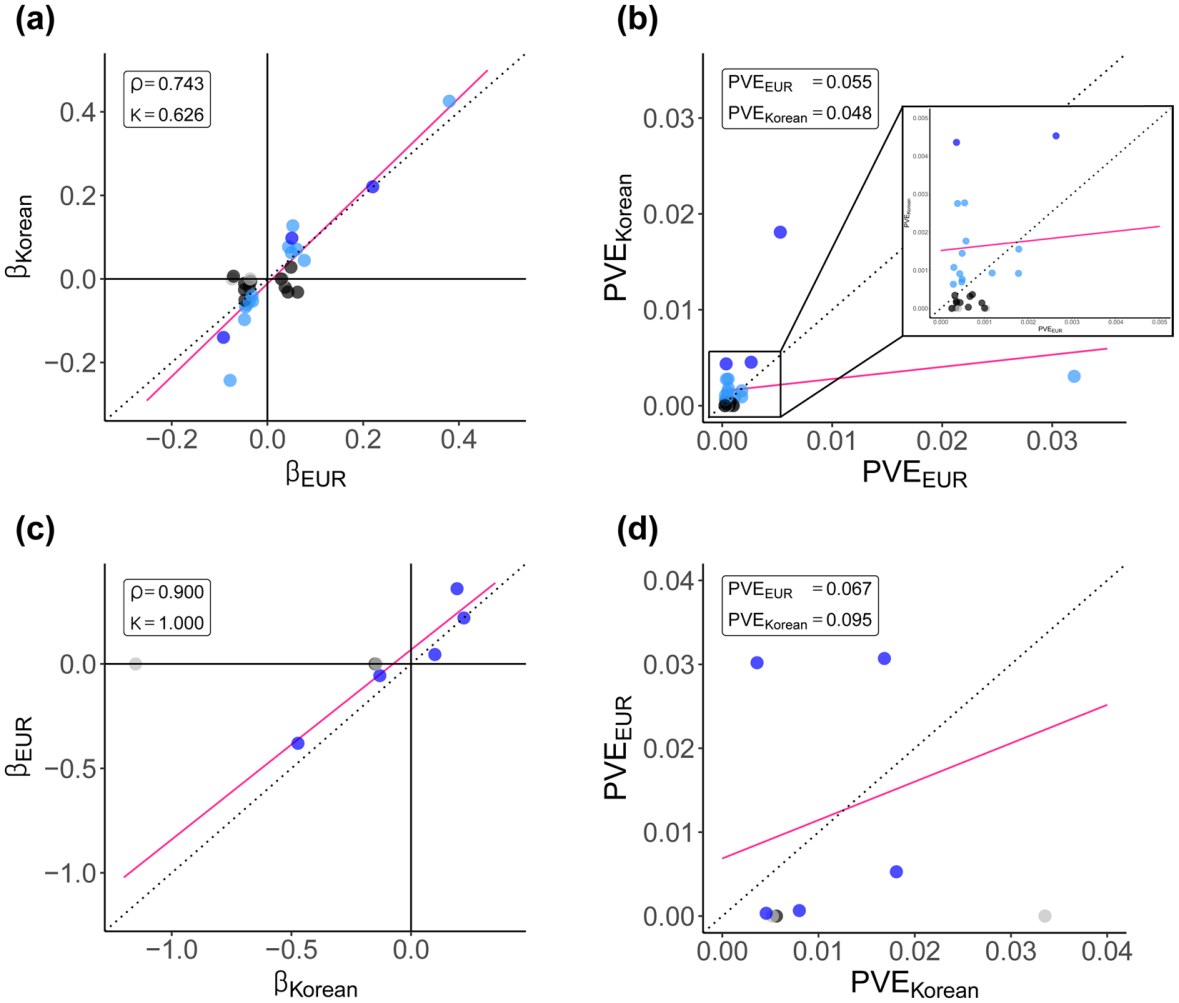
Comparative analysis of effects of and variations in variants identified in the Korean and European cohort studies. (**a**) β coefficients and (**b**) PVE values calculated for GUGC GWAS hit SNPs. (**c**) β coefficients and (**d**) PVE values calculated for lead SNPs identified in our study. Plots indicate high concordance in effect size but different proportion of variance explained by the SNPs identified in our cohort (Korean cohort; *β*_Korean_ and PVE_Korean_) and the GUGC cohort (European cohort; *β*_EUR_ and PVE_EUR_). Each dot indicates a variant and different colours represent the significance level of the variant; genome-wide significance (*P*-value <5 × 10^−8^) in both ethnic cohorts (blue), genome-wide significance in only one cohort but nominal significance in the other (*P*-value <0.05) (sky-blue), insignificance (*P*-value ≥ 0.05) (black) or missing (grey) significance in the other cohort. The regression line (pink) shows the similarity between the two cohorts. There is greater dissimilarity between the two cohorts when the regression line is far from the y = x line (dotted line). Variants missing in any cohort were excluded from the regression analysis. Abbreviations: *ρ*, Spearman’s correlation coefficient; *κ*, Cohen’s kappa coefficient; *β*, coefficient of each SNP obtained by linear regression; *PVE*, proportion of variance in phenotype explained by a given SNP.

Spearman’s correlation coefficient of effect sizes for the GUGC GWAS hit SNPs was 0.743, and it was calculated to be 0.878 for the lead SNPs in our study (Fig. 2a,c). The Cohen’s kappa (*κ*) coefficients of effect sizes were also high (Supplementary Table S2). In contrast, the sum of the PVE values was different between the two cohorts. The sum of PVE for the lead SNPs in our study was larger in Koreans (0.135) than in Europeans (0.071), whereas that for GUGC GWAS hit SNPs was slightly larger in Europeans (0.055) than in Koreans (0.048) (Fig. 2b,d).

### Validation of low-frequency variants and PRSs of common SNPs

**Figure 3:**
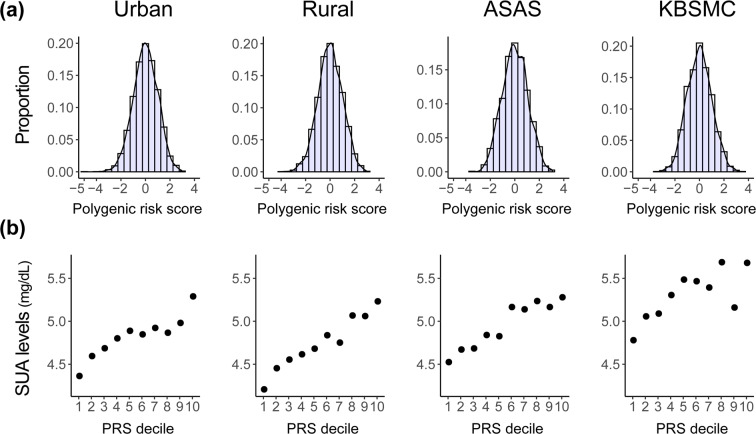
Distribution of polygenic risk scores (PRS) and serum uric acid (SUA) levels. (**a**) Polygenic risk scores corresponding to significantly common SNPs were binned by 0.25. (**b**) Overall pattern of PRS corresponding to the distribution of SUA levels. Participants in each cohort were binned into 10 deciles according to the PRS, and each dot indicates average SUA levels.

The linear regression model that comprised PRSs of common variants associated with SUA levels, the two low-frequency variants, and covariates was fitted to the discovery phase cohorts and validated in the two independent Ansan-Ansung and KBSMC cohorts. The PRSs were normally distributed and the overall distribution correlated with SUA levels (Fig. 3).

The highest and lowest bins of the PRSs corresponded to the highest and lowest levels of SUA, respectively. The PRS was significant in both validation cohorts (*P*-value = 7.06 × 10^−13^, β = 0.24 mg/dL, SE = 0.03 mg/dL in the Ansan-Ansung cohort and *P*-value = 7.93 × 10^−21^, β = 0.21 mg/dL, SE = 0.02 mg/dL in the KBSMC cohort) (Table 3). Among the two low-frequency variants, the p.W258X nonsense mutation of *SLC22A12* showed nominal association (*P*-value = 0.047). There was insufficient data to verify the significance of these low-frequency variants because of the small sample size in the cohort study.

**Table 3:** Estimated coefficients and standard errors obtained on modelling trends in SUA by linear regression.

	SNP	Carriers	EAF	β	SE	L95	U95	*P*-value
Urban (*n* = 3,585)	rs121907892	0/68/3517	0.009	−0.896	0.118	−1.128	−0.665	3.90 × 10^−14^
	rs16890979	0/59/3526	0.008	−0.439	0.124	−0.683	−0.195	4.26 × 10^−4^
	PRS (N_SNPs_ = 14)	not available	not available	0.229	0.016	0.198	0.260	8.95 × 10^−45^
Rural (*n* = 3,296)	rs121907892	1/121/3174	0.018	−0.774	0.093	−0.956	−0.592	1.16 × 10^−16^
	rs16890979	0/61/3235	0.009	−0.446	0.128	−0.697	−0.195	5.03 × 10^−4^
	PRS (N_SNPs_ = 14)	not available	not available	0.281	0.018	0.246	0.316	1.73 × 10^−54^
Ansan-Ansung (*n* = 1,167)	rs121907892	0/21/1146	0.008	−0.489	0.245	−0.970	−0.008	0.047
	rs16890979	0/18/1149	0.011	−0.002	0.264	−0.519	0.514	0.993
	PRS (N_SNPs_ = 11)	not available	not available	0.237	0.033	0.173	0.301	7.06 × 10^−13^
KBSMC (*n* = 2,027)	rs121907892	—	—	—	—	—	—	—
	rs16890979	—	—	—	—	—	—	—
	PRS (N_SNPs_ = 13)	not available	not available	0.211	0.022	0.168	0.255	7.93 × 10^−21^

All covariates used in the association analysis were adjusted for estimating the coefficients and standard errors.

Abbreviations: PRS, standardised polygenic risk scores; N_SNPs_, number of SNPs included in PRS; Carriers, number of carriers (homozygous for effective alleles and heterozygous/homozygous for non-effective alleles); EAF, effective allele frequency; β, coefficient of each mutation or PRS obtained by linear regression; SE, standard error; L95, lower bound of confidence interval of β; U95, upper bound of confidence interval of β.

## Discussion

The main findings of our study include the identification of genome-wide significant association of common and low-frequency variants with SUA levels in Koreans, and the validation of the PRS model of SUA in different cohort studies. The strength of our study is that it includes both urban and rural dwelling participants across Korea and represents an investigation on alcohol consumption. In addition, we have assessed our PRS model in independent cohorts and used available data of genotype-phenotype associations from a large European GWAS for comparison. Our study evaluates the contribution of both low-frequency and common variants to SUA levels by using an HRC-based imputation.

Our results demonstrate the benefits of using a large imputation panel such as the HRC reference panel for discovery, and characterisation of common and low-frequency variants contributing to SUA levels in the general population. We replicated several previously known genetic loci associated with SUA levels (*ABCG2*, *SLC2A9*, *NRXN2*, *NAA25*, *SLC17A3*-*SLC17A2*, *SLC22A12*, and *BCAS3*) reported for the European population and identified independent lead SNPs in two loci (*SLC22A12* and *NAA25*) between the European GWAS and the present study. Comparison with results of the GUGC European study showed that effect sizes of common variants were comparable between East Asian and European populations, whereas allele frequencies and sum of PVE of variants were higher in the population in which the corresponding variants were identified. These results suggest that SUA levels may be affected by population-specific variants as well as shared variants at the same locus, and in particular by population-specific low-frequency variants.

The population-specific *SLC22A12* p.W258X variant and the *SLC2A9* p.W282I variant were accurately imputed (R^2^ ≥ 0.8) using the HRC reference panel. We identified an association between a low-frequency, *SLC22A12* loss-of-function variant (rs121907892) and SUA levels in the general population at a genome-wide significance level *(P*-value <5 × 10^−8^)^9,15,32^. *SLC22A12* p.W258X is a well-known variant for hypouricemia in Koreans^33^. This variant has been earlier identified only by sequencing analysis^20,34^ because of its rarer frequency and weaker linkage disequilibrium with other neighbouring common variants analysed in GWASs. Other common variants in this region were identified in the European population study (rs505802)^15^ and AGEN study (rs504915) including a combined total of ~110,347 Asian individuals^21^. The imputation in AGEN was performed on the basis of the HapMap Phase 2 reference panel. In a more recent analysis by Lee et al., who used 1000 Genomes phase 3 haplotypes as a reference panel for imputation, the founder mutation was still not identified^35^. The observed significant association near the *NAA25* gene on chromosome 12 was previously reported, although different genes (*ALDH2*, *ATXN2*, and *CUX*) were reported for this region. The lead SNP was in high linkage disequilibrium with rs671 in *ALDH2*, a common missense variant in East Asians, but mostly absent in other populations. Our results of alcohol intake-adjusted association analysis and comparison with GWAS for alcohol intake suggests that the association of this region with SUA levels is mediated by alcohol consumption or metabolism through *ALDH2*.

The sex-specific *CDH13* variant (rs8063966) was only detected in females. This may be associated with lower levels of SUA in women since oestradiol and progesterone have been shown to be associated with *CDH13* expression^36^. Estrogen is known to exert a protective effect on SUA levels, with SUA starting to rise in the late-menopausal transition stage^37^. Further research is needed to elucidate the mechanism underlying the interplay of *CDH13* and SUA levels in women.

The missense variant *SLC2A9* p.W282I (rs16890979) has also been previously reported in European and African populations^29^. Renal hypouricemia is believed to arise owing to genetic defects in *SLC22A12* and *SLC2A9*; this is based on the “rare disease, rare variant hypothesis”^38^. In contrast, we tested a regression model of low-frequency variants and PRSs of common alleles associated with SUA levels. Our model showed their independent effects despite predicting a relatively small proportion of variance in SUA levels of the general population, reflecting the “combinational effects of common and rare variants”. Our linear regression model also showed that the SUA levels increased by 0.21–0.28 per 1 standard deviation (SD) increase in PRS and increased by 0.77–0.90 (rs121907892) and 0.44–0.45 (rs16890979) per risk allele of the low-frequent missense variant in the general population.

Our study has several limitations. First, since we enrolled a cohort of ethnically homogeneous Koreans, the generalisability of our results to other populations may be limited. For example, the rare *SLC22A12* p.W258X variant has been found only in the East Asian population, whereas different missense *SLC22A12* variants (c.1245-1253del and p.T467M) have been found in the Roma population with renal hypouricemia^39^. Second, we were not able to analyse all the low-frequency variants due to the lack of sequencing data and population-matched reference panels for imputation. The power to detect additional rare and less frequent disease-causing variants is expected be improve with the availability of reference panels that include sequencing data of more diverse racial/ethnic (e.g., East Asians) samples. Third, we conducted the present analysis in the general population, and therefore, we could not assess the role of genetic variants in people with impaired renal functions. Our results may be different from those reported for gout patients or individuals with impaired renal function, who may exhibit distinct pathways for SUA regulation by other transporters such as *ABCG2*^10,40^. Fourth, potentially confounding environmental factors were not considered in our analysis. A potential contribution of genetic interaction with other factors such as food and drug intake, and chronic disease conditions in the pathophysiology of SUA should be examined. The combination of all the significant genetic loci identified in this study explained approximately 9.55% of the variance in SUA levels of our study, suggesting the need for discovery of additional epigenetic and genetic factors in larger, more comprehensive cohorts.

In summary, we replicated the previously known common genetic variants associated with SUA in the Korean population and identified low-frequency variants that exerted substantial impact on reduced SUA levels. Further studies are needed to identify additional variants associated with SUA and to evaluate our model under different disease conditions (e.g., gout and chronic kidney disease).

## Methods

### Ethics, consent, and permissions

This study was approved by the institutional review board of Sungkyunkwan University (IRB# SKKU 2017-12-007) and Kangbuk Samsung Hospital (KBSMC 2013-01-245-008), and written informed consent was obtained from all participants. All experiments were performed in accordance with all applicable institutional and governmental regulations concerning the ethical use of human participants.

### Study participants

The Korean Genome and Epidemiology Study (KoGES) is a national biobank for genomic and epidemiological studies^41^. The discovery-phase samples comprised two cohorts from KoGES, namely a nation-wide cohort from urban (3,585 individuals) and another from rural (3,296 individuals) areas. In total, 6,881 individuals with no missing covariates were included in the present study. Genotyping was conducted on the Affymetrix 6.0 and the Illumina Omni1 arrays for the urban and rural cohorts, respectively. We evaluated two low-frequency variants and PRS for validation in 1,167 individuals of a community-based KoGES cohort from Ansan and Ansung areas, in which SUA levels were measured, and in 2,027 individuals from the Health Screening and Examination cohort of the Kangbuk Samsung Hospital (KBSMC). Genotyping was conducted using the Affymetrix 5.0 array and Illumina HumanCore BeadChips for the Ansan-Ansung and KBSMC cohorts, respectively.

### Quality control, imputation, and annotation

Sample-level and SNP-level quality controls were performed on the genotyped data using PLINK 1.9^42^. SNPs with MAF < 1%, call rate <98%, and deviation from Hardy-Weinberg equilibrium (*P*-value <1.0 × 10^−6^) were excluded. Samples were excluded based on criteria including call rate <95%, heterozygosity rate (samples with observed heterozygosity rate 3 SD away from the mean were removed), and principal components derived from the genome-wide genotype data (samples with the first and second principal components 3 SD away from the mean were removed). We excluded one of related pairs of individuals with second-degree or closer relationships using KING 2.1^43^. After quality control, the genotype data was phased using Eagle 2.3^44^ and imputed on the reference panel of the Haplotype Reference Consortium (HRC r1.1 2016) using Minimac3^45^. SNPs with low imputation quality (R^2^ < 0.8) and MAF < 0.5% were excluded from the main analysis. We annotated gene symbols closest to each SNP using ANNOVAR^46^; the two nearest genes were annotated for intergenic SNPs.

### Association analysis

The association of SNPs with SUA was tested using a linear regression model adjusted for sex, age, body mass index (BMI), estimated glomerular filtration rate (eGFR), diabetes, hypertension, hyperlipidaemia, systolic blood pressure, total cholesterol, high-density lipoprotein (HDL) cholesterol, and triglycerides. Blood pressure medication was measured in the rural cohort and adjusted in linear regression. The equation from the Chronic Kidney Disease Epidemiology collaboration (CKD-EPI) was used for the estimation of eGFR^47^. We performed an inverse variance-weighted fixed-effects meta-analysis of SUA levels to combine the summary statistics of the association test results from the two discovery cohorts using METAL^48^. The meta-analysis results were double-genomic-controlled for population structure. In the meta-analysis, SNPs that reached a level of genome-wide significance (*P*-value <5.0 × 10^−8^) for association with the SUA levels were considered significant. For nonsynonymous variants, Bonferroni’s correction threshold (*P*-value <4.31 × 10^−6^, 0.05 for a total of 11,600 nonsynonymous tested variants) was used.

### Conditional analysis

We performed conditional association analysis for each significant locus using GCTA-COJO^49^ to identify statistically independent SNPs, which have been listed in Table 2. The peak SNPs in each significant locus were selected as the primary associated lead SNP and the association analysis conditioning on the primary lead SNP was conducted for variants within the surrounding 2-megabase pairs region. If there were significant SNPs with a conditional *P*-value that reached a level of significance described in the association analysis, the peak SNP of conditional analysis was selected as the secondary associated lead SNP. The association analysis conditioning on the primary and secondary lead SNPs was conducted for variants in the surrounding region. This procedure was repeated until there were no more SNPs that reached the significant level in each locus.

### Alcohol intake

We observed a high linkage disequilibrium between a lead SNP on chromosome 12 and a known functional missense variant (rs671) in the aldehyde dehydrogenase 2 (*ALDH2*) gene. Therefore, alcohol intake-adjusted association of SNPs with SUA was tested using a linear regression model adjusted for sex, age, the first 10 principal components derived from the genome-wide genotype data, and dummy variables for the alcohol intake groups. Several alcohol-related variables were investigated in the urban and rural cohorts, including the prevalence of a drinking habit, average intake per drink by type of alcoholic beverage, and average number of intakes per year. We calculated the daily alcohol intake based on these variables using the following equation:

1 $$Alcohol\,intake(g/day)=\sum \{(Alcohol\,content\,ratio\,)\times (Average\,intake\,per\,drink)\times (Average\,number\,of\,intake\,per\,year)\div365\}$$

The study participants in each cohort were then divided into four groups based on the calculated daily alcohol intake: non-drinker, light drinker (<20 g/day), moderate-heavy drinker (≥ 20 g/day and <50 g/day), and heavy drinker (≥50 g/day). To compare the GWAS results of SUA, we also performed the association analysis of alcohol intake (non-drinkers vs. moderate-heavy or heavy drinkers) using a logistic regression model adjusted for sex, age, and the first 10 principal components.

### Comparison with results from the European cohort study

Summary statistics results from the GUGC were used for comparison of significant associations identified in our study with those reported for European populations. We extracted lead SNPs from the present study and GWAS hit SNPs from GUGC. Three of the GUGC GWAS hit SNPs were not found in our datasets and nine of the lead SNPs in our study were absent from the GUGC data. We set the β coefficients of these SNPs to zero. To compare the effect sizes from our meta-analysis with those of GUGC summary statistics, we used Cohen’s kappa (*κ*) coefficient to determine the direction of effect size and Spearman’s correlation coefficient (*ρ*). In addition, we used proportion of variance in phenotype explained by a given SNP (PVE) for comparison. The GUGC summary statistics provides sample size, MAF, effect size (β coefficient), and SE of effect size for each SNP above the *P*-value <5.0 × 10^−8^ threshold^9^. We estimated PVE values using the equation below^50^:

2 $$PVE=\frac{2{\hat{\beta }}^{2}MAF(1-MAF)}{2{\hat{\beta }}^{2}MAF(1-MAF)+{(se(\hat{\beta }))}^{2}2NMAF(1-MAF)}$$

we used values of the MAF of Europeans from the Genome Aggregation Database (gnomAD) because information on allele frequency was not included in the GUGC summary statistics^51^. Comparative measurements were calculated after excluding the variants that were missing in our meta-analysis result or from the GUGC summary statistics.

### Polygenic risk scoring and model construction

We calculated PRS values as a weighted sum of the effects multiplied by the number of alleles based on the β estimates for SUA-raising alleles for 14 common (MAF ≥ 5%) SNPs that reached a level of genome-wide significance (*P* < 5.0 × 10^−8^) for association with SUA levels and were not in a high linkage disequilibrium (*r*^2^ ≤ 0.2) with the other variants. The PRS of β estimates for SUA-raising alleles was standardised to zero mean and unit variance. For nonsynonymous variants, Bonferroni’s correction threshold (*P* < 4.31 × 10^−6^, 0.05 for a total of 11,600 nonsynonymous variants) was applied to correct for the multiple testing problem. Two low-frequency SNPs (MAF ~1%), rs121907892 and rs16890979, which passed the threshold were also included in the linear regression model-based analysis of SUA levels. We constructed a linear regression model on SUA levels that comprised these PRSs and two low-frequency nonsynonymous variants. The linear regression model was adjusted for the following covariates available in all cohorts for validation: sex, age, BMI, eGFR, diabetes, hypertension, hyperlipidaemia, systolic blood pressure, total cholesterol, HDL cholesterol, and triglycerides.

## Supplementary information

Supplementary Tables and Figures.

Supplementary Table S1.
