# Supplementary material for: Polygenic analysis of the effect of common and low-frequency genetic variants on serum uric acid levels in Korean individuals
Source: Sci Rep. 2020 Jun 8;10:9179. doi: 10.1038/s41598-020-66064-z (PMC7280503; doi:10.1038/s41598-020-66064-z)

Polygenic analysis of the effect of common and low-frequency genetic variants on serum uric acid levels in Korean individuals

Sung Kweon Cho, MD, PhD[1,2,#], Beomsu Kim, BS[1,#], Woojae Myung, MD, PhD[3], Yoosoo Chang, MD, PhD[4], Seungho Ryu, MD, PhD[4], Han-Na Kim, PhD[4,5], Hyung-Lae Kim, MD, PhD[6], Po-Hsiu Kuo, PhD[7], Cheryl A. Winkler, PhD[2,*], Hong-Hee Won, PhD[1,*]

[1]Samsung Advanced Institute for Health Sciences and Technology (SAIHST), Sungkyunkwan University, Samsung Medical Center, Seoul, Republic of Korea

[2]Molecular Genetic Epidemiology Section, Basic Research Laboratory, Center for Cancer Research, National Cancer Institute, Frederick, MD, USA

[3]Department of Neuropsychiatry, Seoul National University Bundang Hospital, Seongnam-si, Republic of Korea

[4]Center for Cohort Studies, Total Healthcare Center, Kangbuk Samsung Hospital, Sungkyunkwan University School of Medicine, Seoul, Republic of Korea

[5]Medical Research Institute, Kangbuk Samsung Hospital, Sungkyunkwan University School of Medicine, Seoul, Republic of Korea

[6]Department of Biochemistry, Ewha Womans University, Seoul, Republic of Korea

[7]Department of Public Health & Institute of Epidemiology and Preventive Medicine, College of Public Health, National Taiwan University, Taiwan

[#]These authors contributed equally to this work

Running head: Effect of common and low-frequency genetic variants on serum uric acid

*Address correspondence to:

Hong-Hee Won, PhD

Samsung Advanced Institute for Health Sciences and Technology (SAIHST), Sungkyunkwan University, Samsung Medical Center, 81 Irwon-ro, Gangnam-gu, Seoul 06351, Republic of Korea.

Tel: +82-2-2148-7566

Fax: +82-2-3410-0534

E-mail: wonhh@skku.edu

Cheryl A. Winkler, PhD

National Cancer Institute ATRF, D-3024, 8560 Progress Drive, Frederick, Maryland 21701, USA.

Tel: +1-301-846-5747

Fax: +1-301-846-1767

E-mail: winklerc@mail.nih.gov

Supplementary Materials

Table S1. All SNPs associated with SUA corresponding to *P*-values < 5.0 × 10^{-8}.

Excel file

Table S2. Cohen's kappa (*κ*) coefficients. Cohen's kappa coefficients and a contingency table of the sign of β coefficients are shown below. The values listed in contingency table represent the count of β coefficients, which can have either a negative or a positive sign. Missing variants were excluded from this calculation. (**a**) Lead SNPs in our study (*κ* = 1.000). (**b**) All the GUGC GWAS hit SNPs (κ = 0.626). (**c**) GUGC GWAS hit SNPs excluding SNPs that are not significant (*P*-value ≥ 0.05) identified from our study (*κ* = 1.000).

(**a**) Lead SNPs in our study (*κ* = 1.000).

		Our study	
		Negative	Positive
GUGC	Negative	2	0
	Positive	0	3

(**b**) All the GUGC GWAS hit SNPs (κ = 0.626).

		GUGC	
		Negative	Positive
Our study	Negative	13	4
	Positive	1	9

(**c**) GUGC GWAS hit SNPs excluding SNPs that are not significant (*P*-value ≥ 0.05) identified from our study (*κ* = 1.000).

		GUGC	
		Negative	Positive
Our study	Negative	8	0
	Positive	0	8

Table S3. Nonsynonymous variants associated with SUA identified from meta-analysis of GWASs.

										EAF in each cohort			
SNP	Chr	BP	Reference gene	Function	A1	A2	β	SE	P-value	Urban	Rural	Ansan-Ansung	KBSMC
rs121907892	11	64361219	*SLC22A12*	nonsense	A	G	-1.151	0.075	7.43×10^{-54}	0.009	0.018	0.008	-
rs2231142	4	89052323	*ABCG2*	missense	T	G	0.221	0.020	2.06×10^{-29}	0.273	0.258	0.266	0.255
rs3733591	4	9922130	*SLC2A9*	missense	T	C	-0.136	0.019	1.30×10^{-12}	0.697	0.706	0.695	0.707
rs2231137	4	89061114	*ABCG2*	missense	T	C	-0.125	0.020	2.53×10^{-10}	0.269	0.262	0.256	0.261
rs671	12	112241766	*ALDH2*	missense	A	G	-0.146	0.024	1.34×10^{-09}	0.153	0.167	0.167	0.156
rs1165196	6	25813150	*SLC17A1*	missense	A	G	0.144	0.025	8.98×10^{-09}	0.867	0.853	0.858	0.856
rs2276961	4	10022981	*SLC2A9*	missense	T	C	0.095	0.018	5.16×10^{-08}	0.456	0.454	0.448	0.452
rs16890979	4	9922167	*SLC2A9*	missense	T	C	-0.473	0.095	5.86×10^{-07}	0.008	0.009	0.011	-
rs12362011	11	65547455	*AP5B1*	missense	A	C	-0.096	0.021	2.94×10^{-06}	0.237	0.248	0.230	0.230
rs7969300	12	111993712	*ATXN2*	missense	T	C	-0.081	0.018	3.30×10^{-06}	0.541	0.549	0.538	0.541

Abbreviations: *Chr*, chromosome number; *BP*, base position; *A1*, effective allele; *A2*, non-effective allele; *EAF*, effective allele frequency; *β*, coefficient of each SNP in linear regression; *SE*, standard error

Table S4. Coefficients and standard errors estimated by the linear regression model of SUA with simple covariates. Age, sex, and the first 10 principal components derived from the genome-wide genotype data were adjusted for estimating the coefficients and standard errors.

	SNP	Carriers	EAF	β	SE	L95	U95	*P*-value
Urban (*n* = 3,585)	rs121907892	0/68/3517	0.009	-0.893	0.132	-1.123	-0.604	7.83×10^{-11}
	rs16890979	0/59/3526	0.008	-0.377	0.140	-0.650	-0.104	6.92×10^{-3}
	PRS (N_{SNPs} = 14)	not available	not available	0.227	0.018	0.191	0.262	3.03×10^{-35}
Rural (*n* = 3,296)	rs121907892	1/121/3174	0.018	-0.834	0.101	-1.033	-0.635	2.94×10^{-16}
	rs16890979	0/61/3235	0.009	-0.431	0.140	-0.705	-0.156	2.10×10^{-3}
	PRS (N_{SNPs} = 14)	not available	not available	0.281	0.019	0.243	0.319	4.58×10^{-46}
Ansan-Ansung (*n* = 1,167)	rs121907892	0/21/1146	0.008	-0.441	0.273	-0.976	0.095	0.107
	rs16890979	0/18/1149	0.011	-0.003	0.294	-0.579	0.573	0.992
	PRS (N_{SNPs} = 11)	not available	not available	0.231	0.037	0.159	0.303	3.76×10^{-10}
KBSMC (*n* = 2,027)	rs121907892	-	-	-	-	-	-	-
	rs16890979	-	-	-	-	-	-	-
	PRS (N_{SNPs} = 13)	not available	not available	0.207	0.024	0.160	0.254	2.18×10^{-17}

Abbreviations: *PRS,* standardised polygenic risk scores; N_{SNPs}, number of SNPs included in PRS calculation; *Carriers,* number of carriers (homozygous for effective alleles and heterozygous/homozygous for non-effective alleles); *EAF*, effective allele frequency; *β*, coefficient of each mutation or PRS in linear regression; *SE*, standard error; *L95*, lower bound of confidence interval of β; *U95*, upper bound of confidence interval of β

Table S5. Description of reference genes of the lead variants. The COSMIC Cancer Gene Census association via MalaCards is listed as a related disease in the "Associated diseases " column. The third column indicates whether the association with SUA levels has been reported in previous GWASs. In cases where the association with SUA has not been previously reported, other reported phenotypes were specified.

Gene	Associated diseases	Association with SUA reported in previous GWASs?
SLC22A12	Hypouricemia, Renal, 1 (RHUC1)	Yes
ABCG2	Blood Group, Junior System (JR)<br>Uric Acid Concentration, Serum, Quantitative Trait Locus 1 (GOUT1)	Yes
SLC2A9	Hypouricemia, Renal, 2 (RHUC2)<br>Hypouricemia, Renal, 1 (RHUC1)	Yes
NRXN2	Spinal Cord Glioma	Yes
NAA25	Shwachman-Diamond Syndrome 1	No; platelet; blood pressure; alcohol drinking; type I diabetes
SLC17A3	Uric Acid Concentration, Serum, Quantitative Trait Locus 4 (GOUT4)	Yes
SLC17A2	-	No; systemic lupus erythematosus; schizophrenia; autism spectrum disorder
BCAS3	Breast cancer (BC)	No; heel bone mineral density; glomerular filtration rate; serum creatinine measurement

Fig. S1. Quantile-quantile (QQ) plots of association results (λ = 0.998). The negative logarithm of the observed (y axis) and the expected (x axis) P-values is plotted for each SNP, and the red line indicates the null hypothesis of no true association.

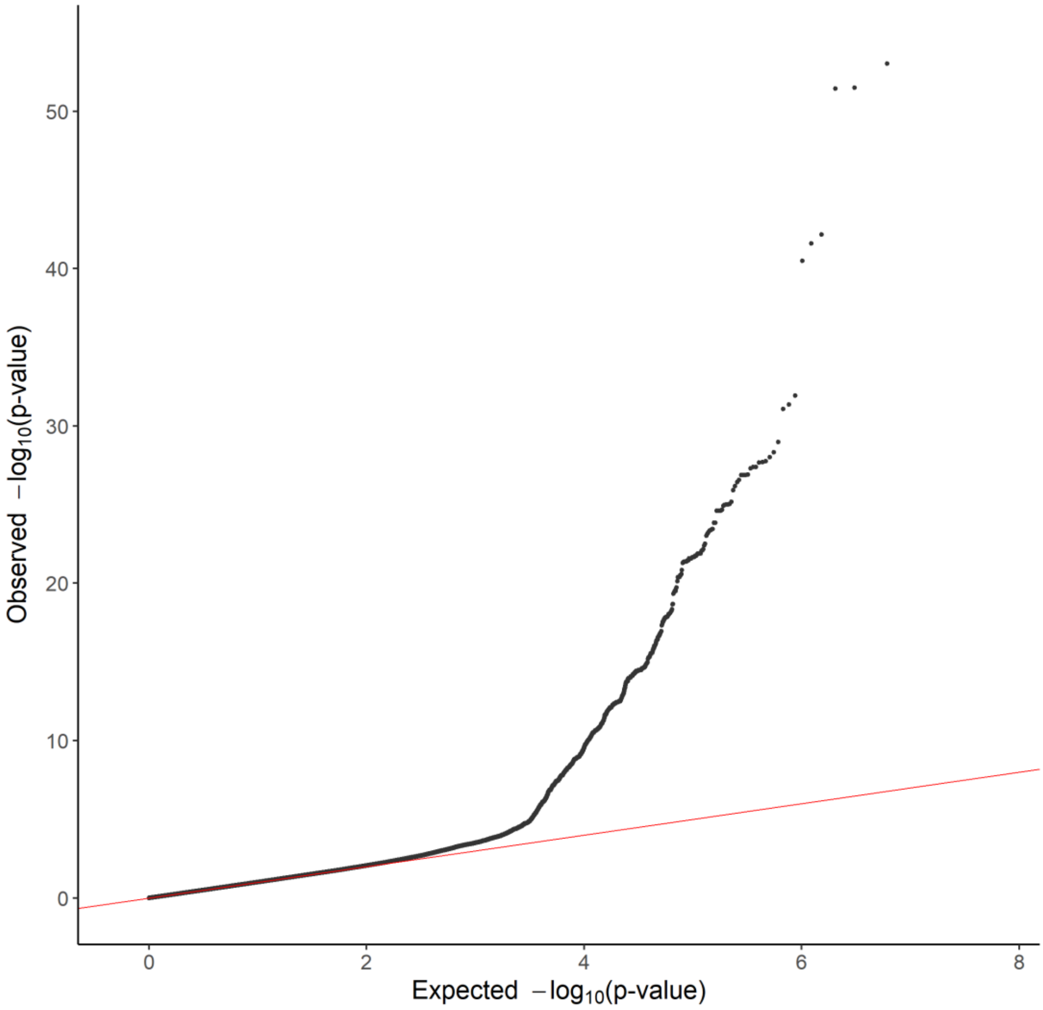

Fig. S2. Regional plots of association for the two low-frequency variants (*SLC22A12* and *SLC2A9*). Variants on (**a**) chromosome 11 (*SLC22A12*) and (**b**) chromosome 4 (*SLC2A9*) associated with SUA. The results of linear regression analysis are shown in purple for the 2-megabase pair region surrounding the low-frequency, nonsynonymous SNP. Each dot indicates a variant and different colours represent the linkage disequilibrium (r^2) of each variant with the low-frequency, nonsynonymous SNP.

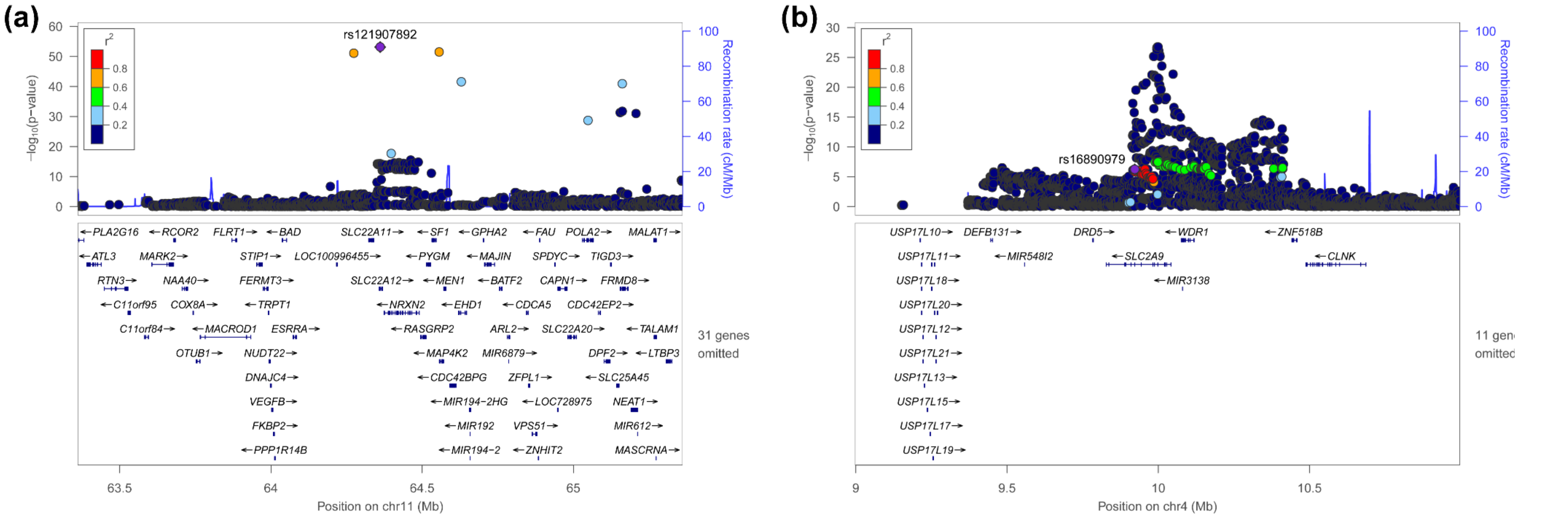

Fig. S3. Regional plots of association for common variants. Results from the logistic regression analysis are shown for the 2-megabase pair region surrounding the peak SNP (in purple). Each dot indicates a variant and different colours represent linkage disequilibrium (r^2) of each variant with the peak SNP. Variants on (**a**) chromosome 4 (*ABCG2*), (**b**) chromosome 4 (*SLC2A9*), (**c**) chromosome 12 (rs116873087), (**d**) chromosome 12 (rs7969300), (**e**) chromosome 6, and (**f**) chromosome 17 associated with SUA. The different colours represent the linkage disequilibrium (r^2) of each variant with the common, nonsynonymous SNP on the *ATXN2* gene.

(**a**) Variants on chromosome 4 (the *ABCG2*) associated with SUA.

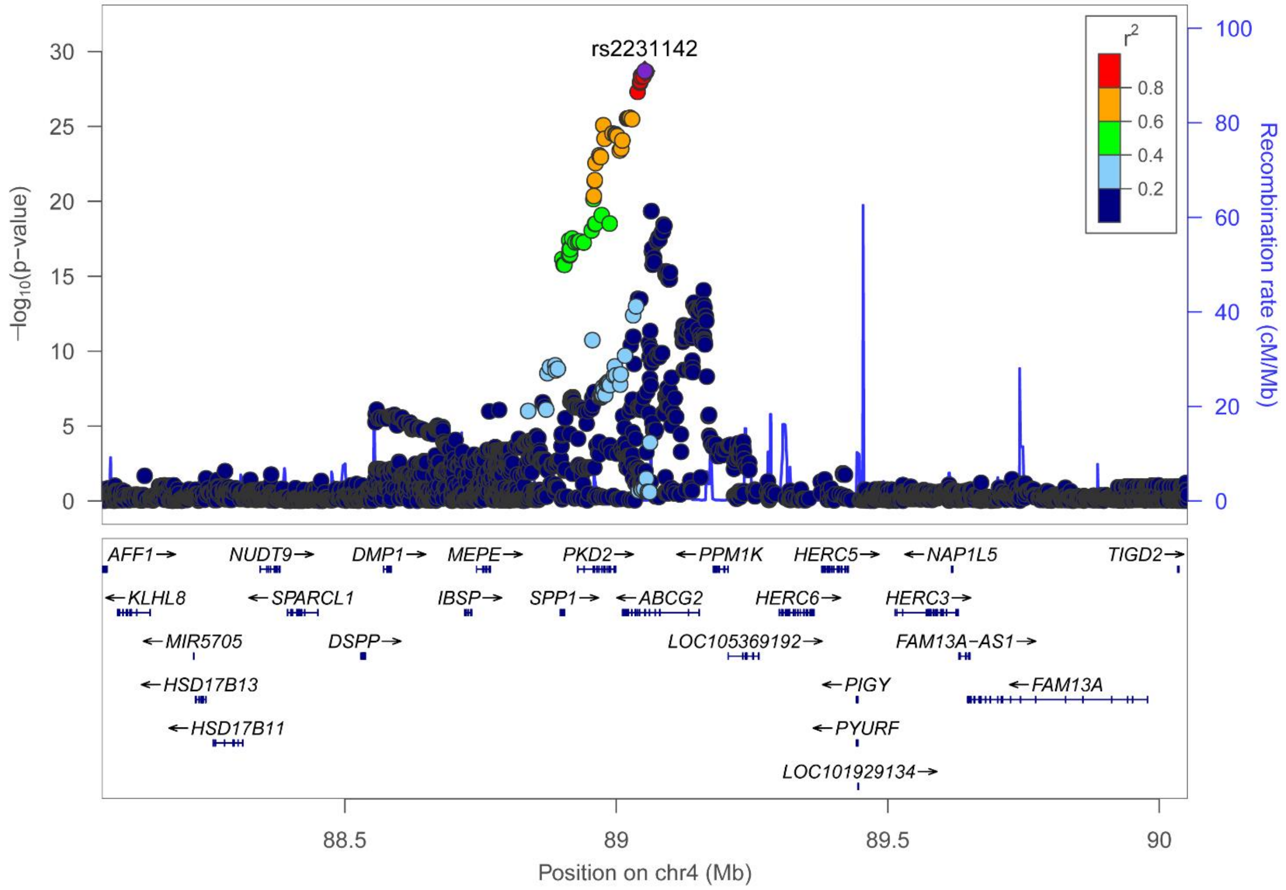

(**b**) Variants on chromosome 4 (the *SLC2A9*) associated with SUA.

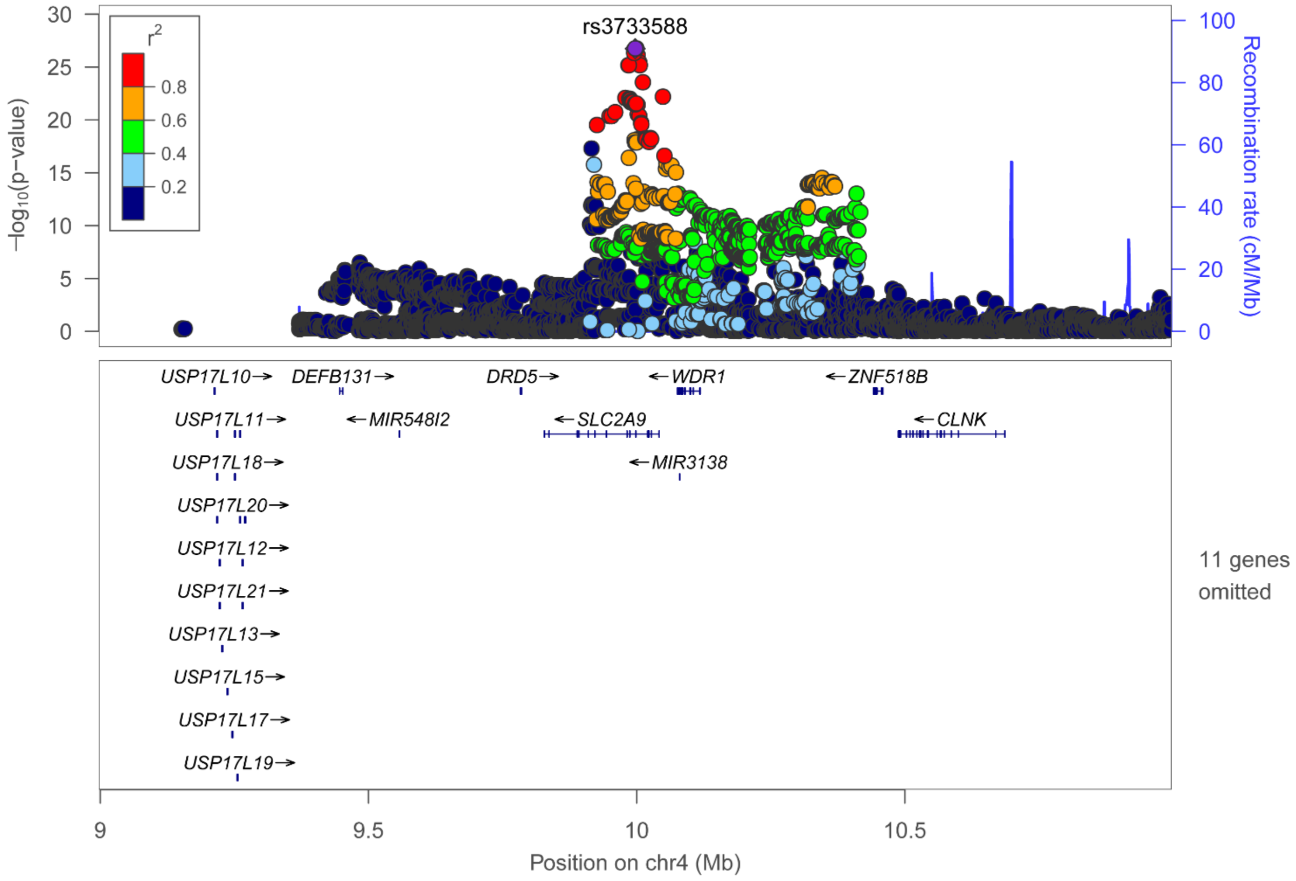

(**c**) Variants on chromosome 12 associated with SUA.

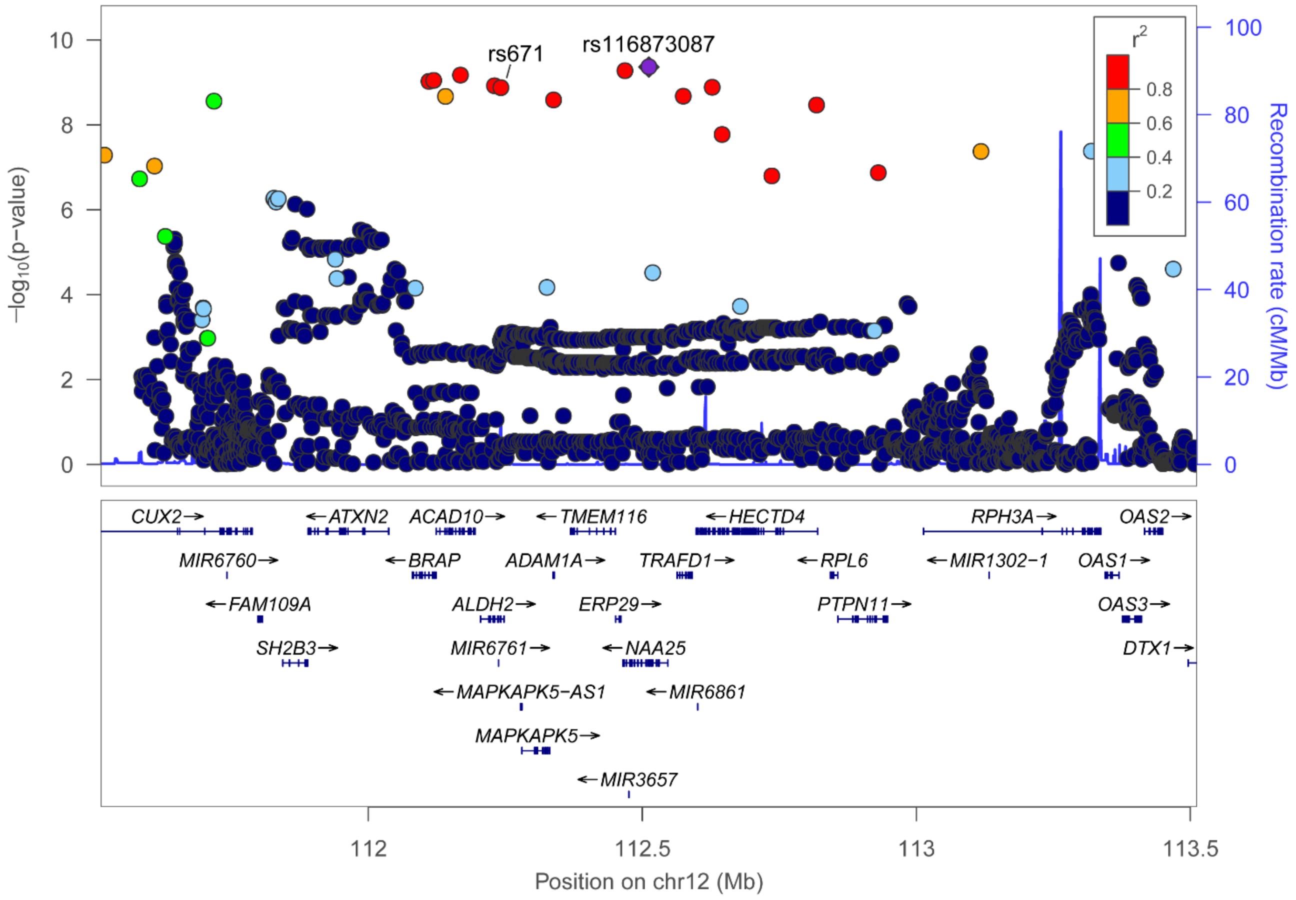

(**d**) Variants on chromosome 12 associated with SUA; different colours represent the linkage disequilibrium (r^2) of each variant with the common, nonsynonymous SNP (rs7969300) on the *ATXN2* gene.

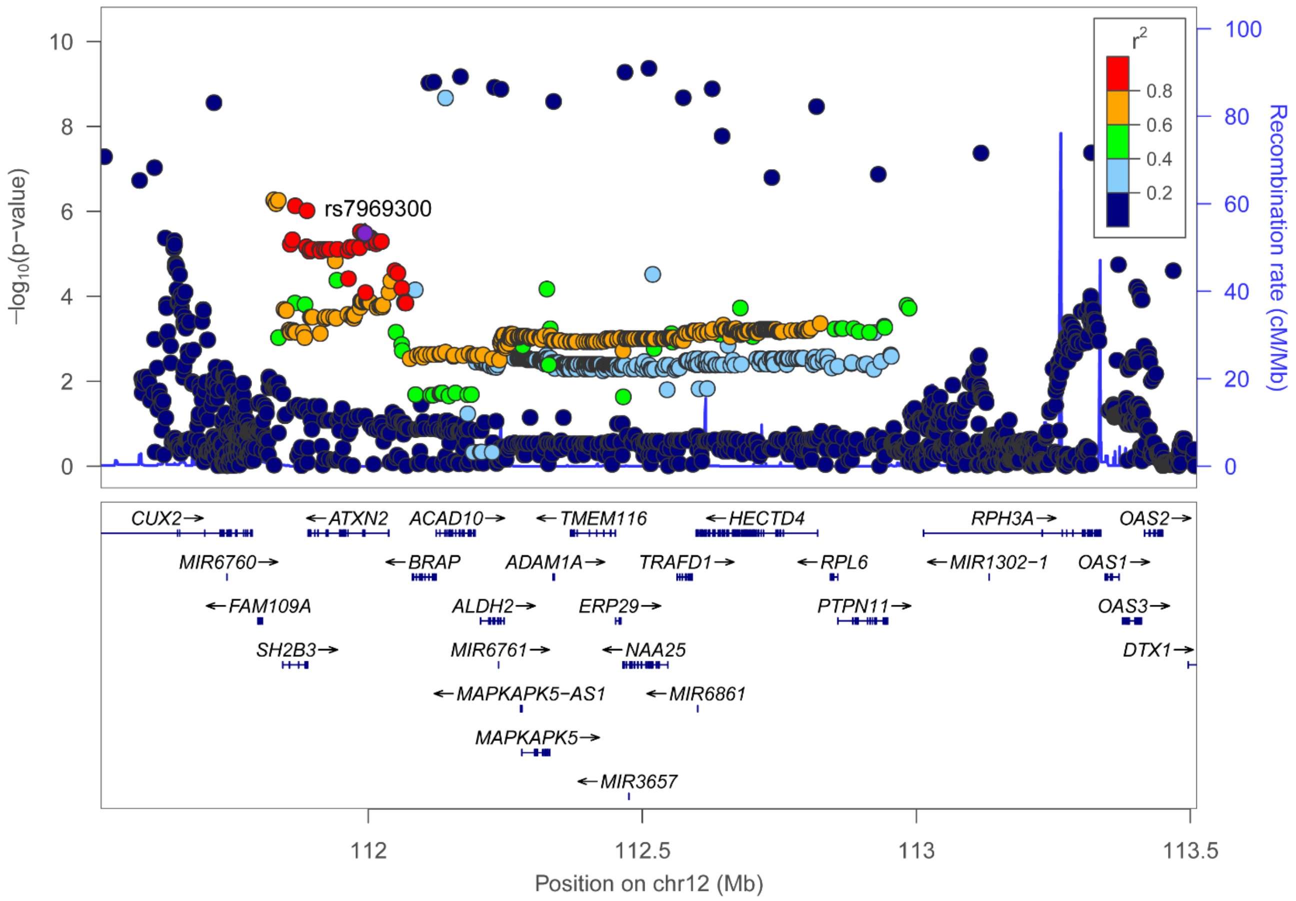

(**e**) Variants on chromosome 6 associated with SUA.

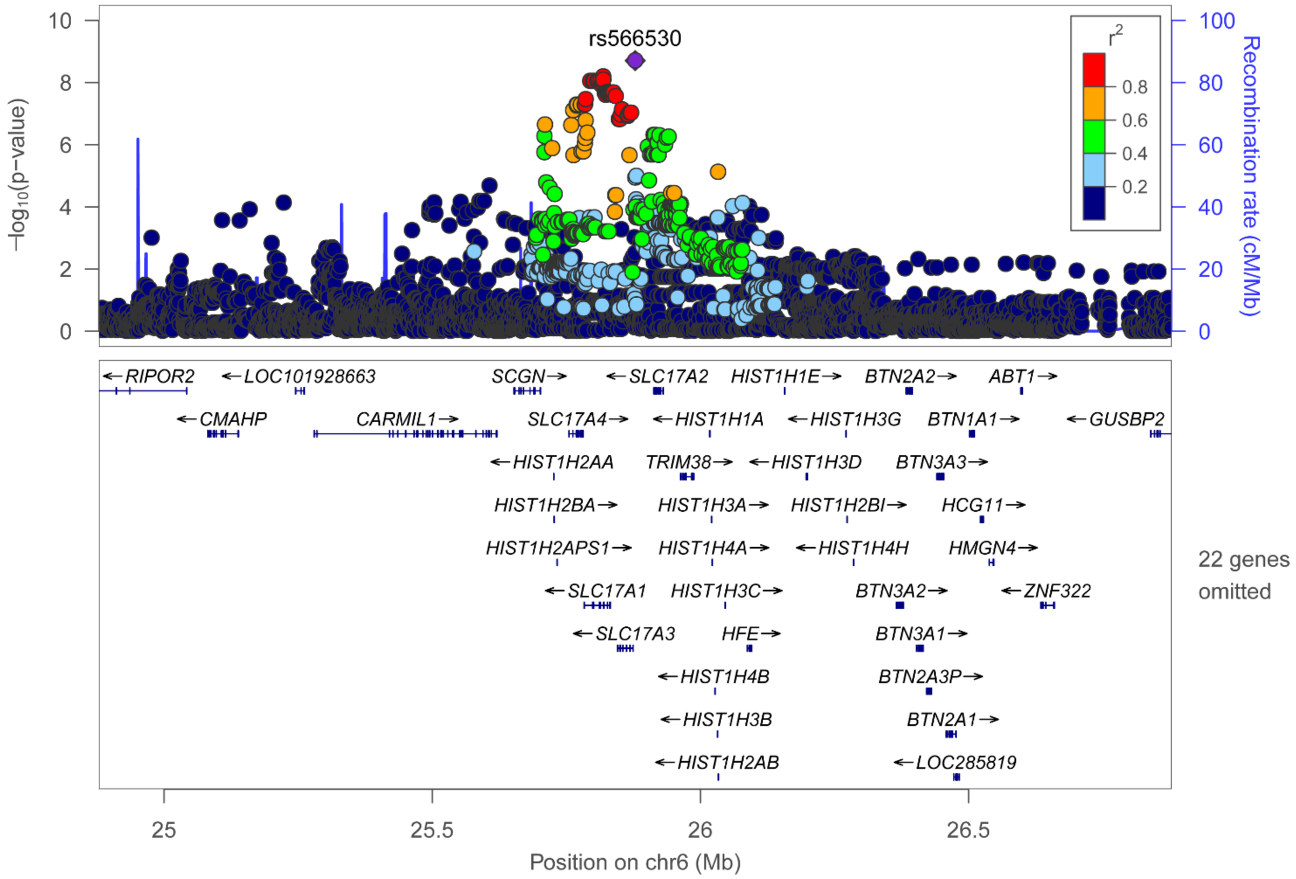

(**f**) Variants on chromosome 17 associated with SUA.

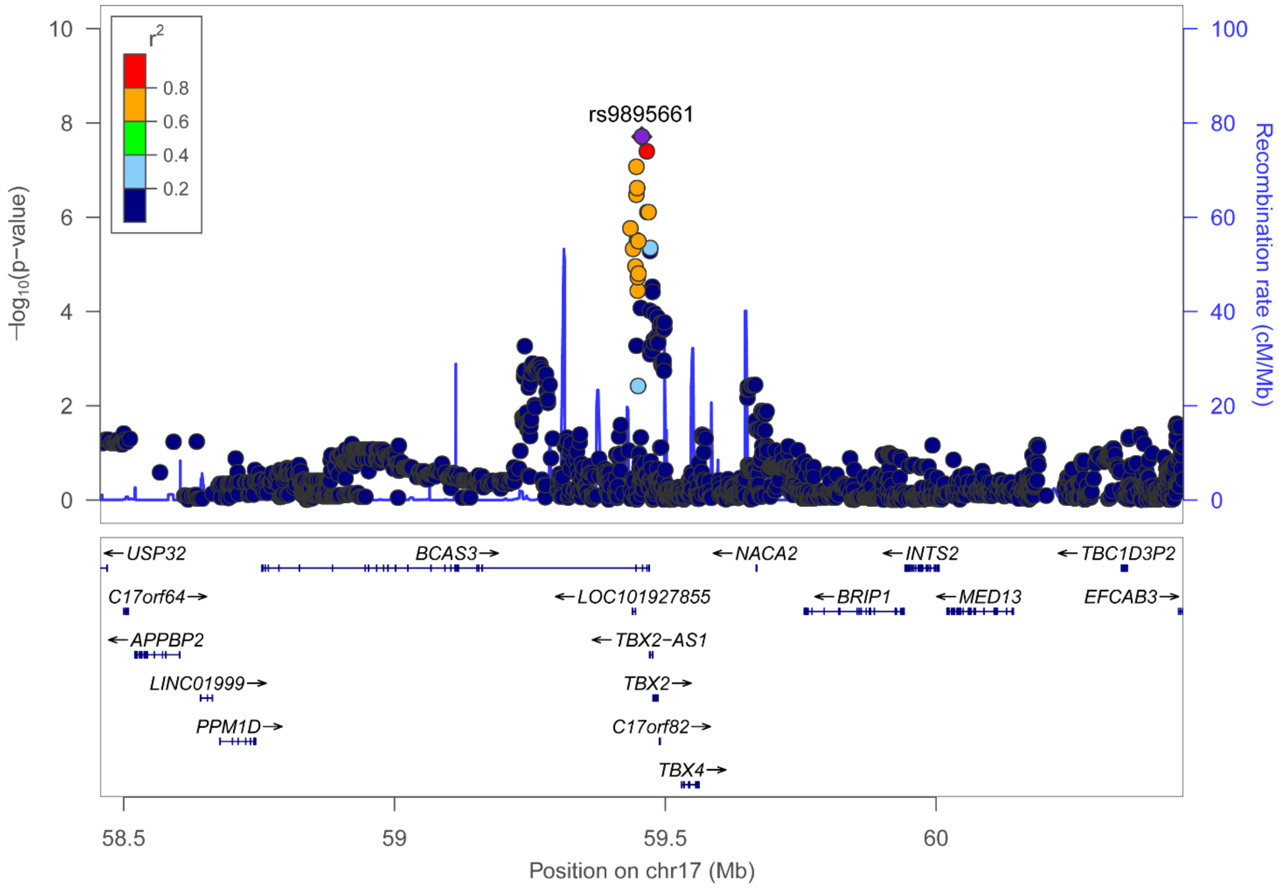

Fig. S4. Regional plots of conditional analysis. The results of conditional analysis for the 2-megabase pair region surrounding the peak SNP are shown in purple. Each dot indicates a variant and different colours represent linkage disequilibrium (r^2) of each variant with the peak SNP. Variants on (**a**) chromosome 11, (**b**) chromosome 4 (*ABCG2*), (**c**) chromosome 4 (*SLC2A9*), (**d**) chromosome 12, (**e**) chromosome 6, and (**f**) chromosome 17 associated with SUA.

(**a**) Variants on chromosome 11 associated with SUA.

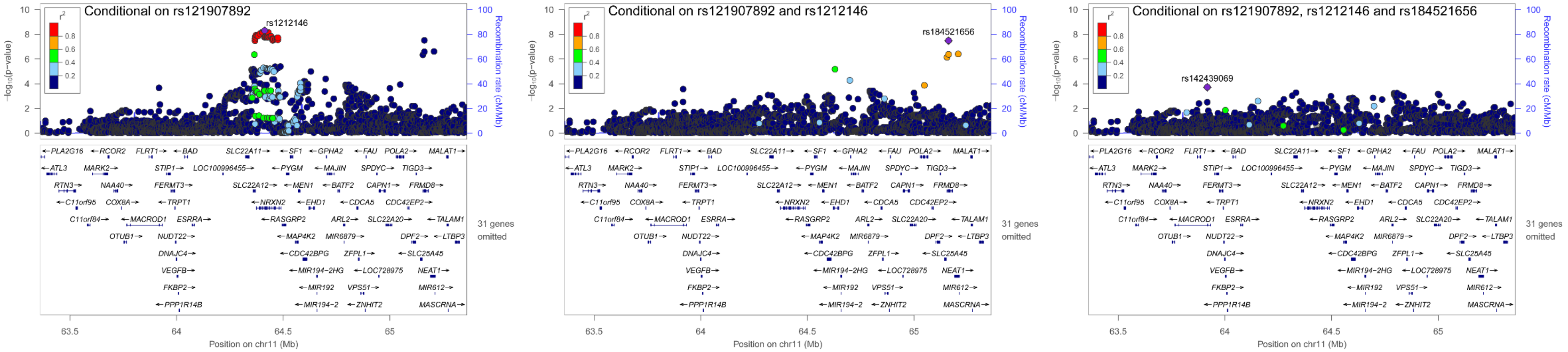

(**b**) Variants on chromosome 4 (the *ABCG2*) associated with SUA.

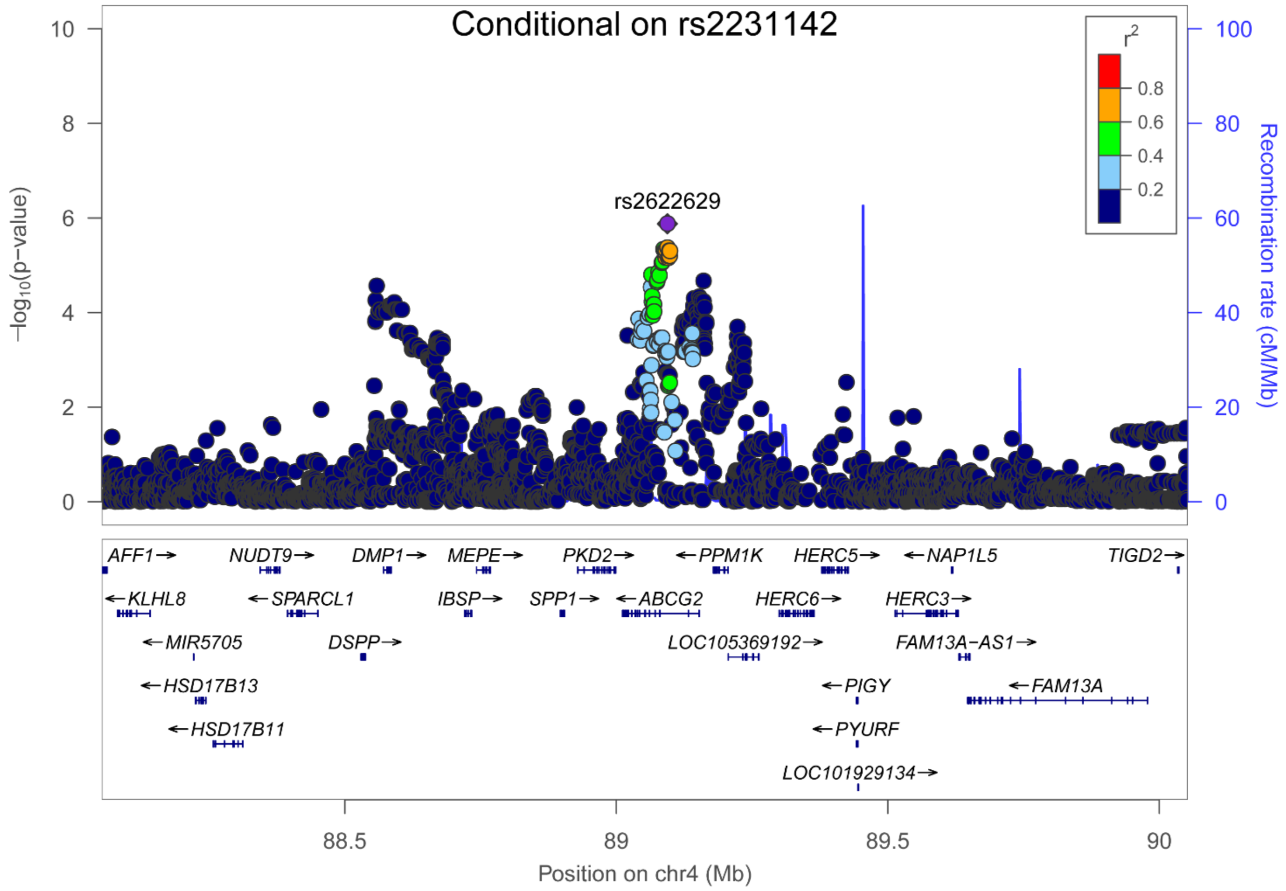

(**c**) Variants on chromosome 4 (the *SLC2A9*) associated with SUA.

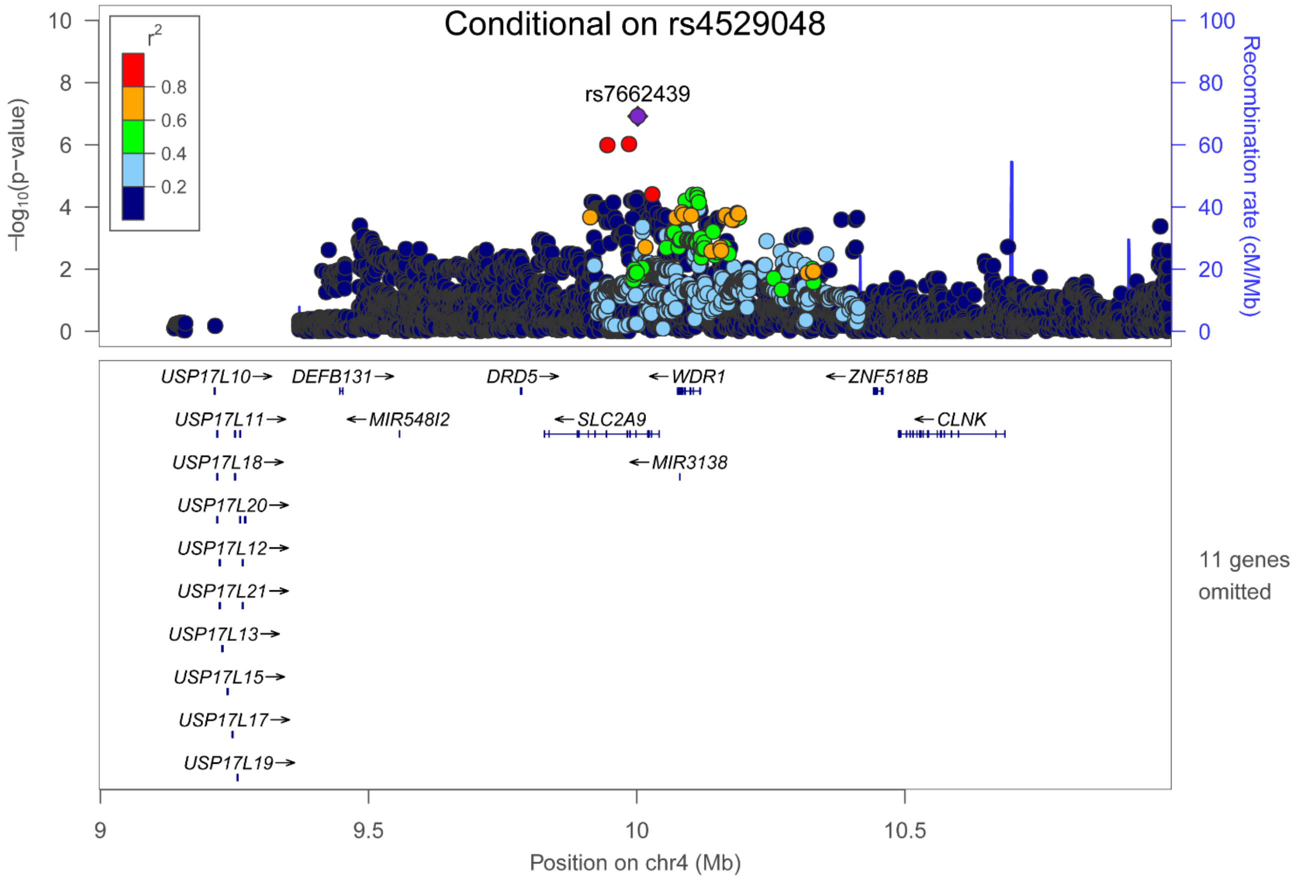

(**d**) Variants on chromosome 12 associated with SUA.

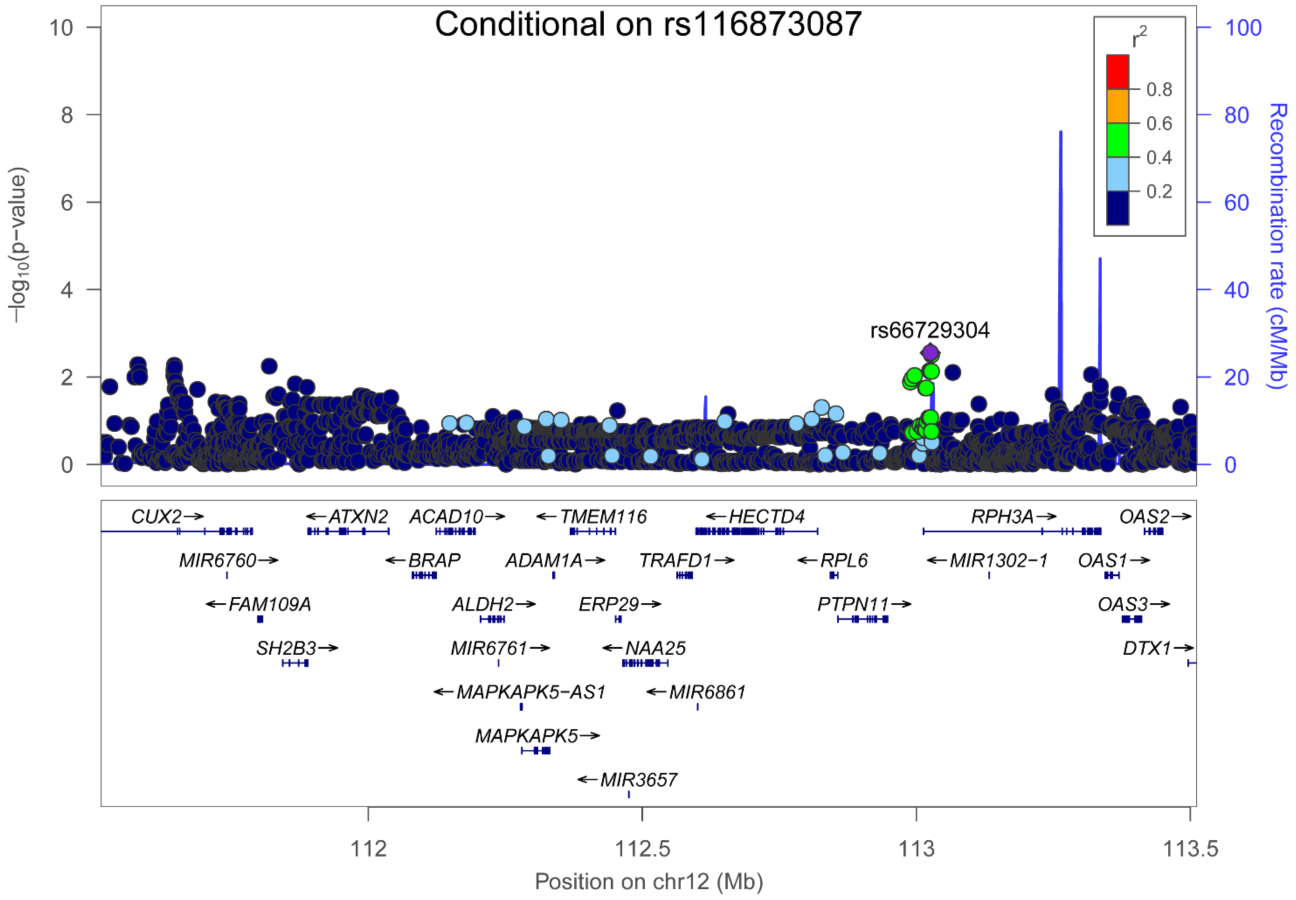

(**e**) Variants on chromosome 6 associated with SUA.

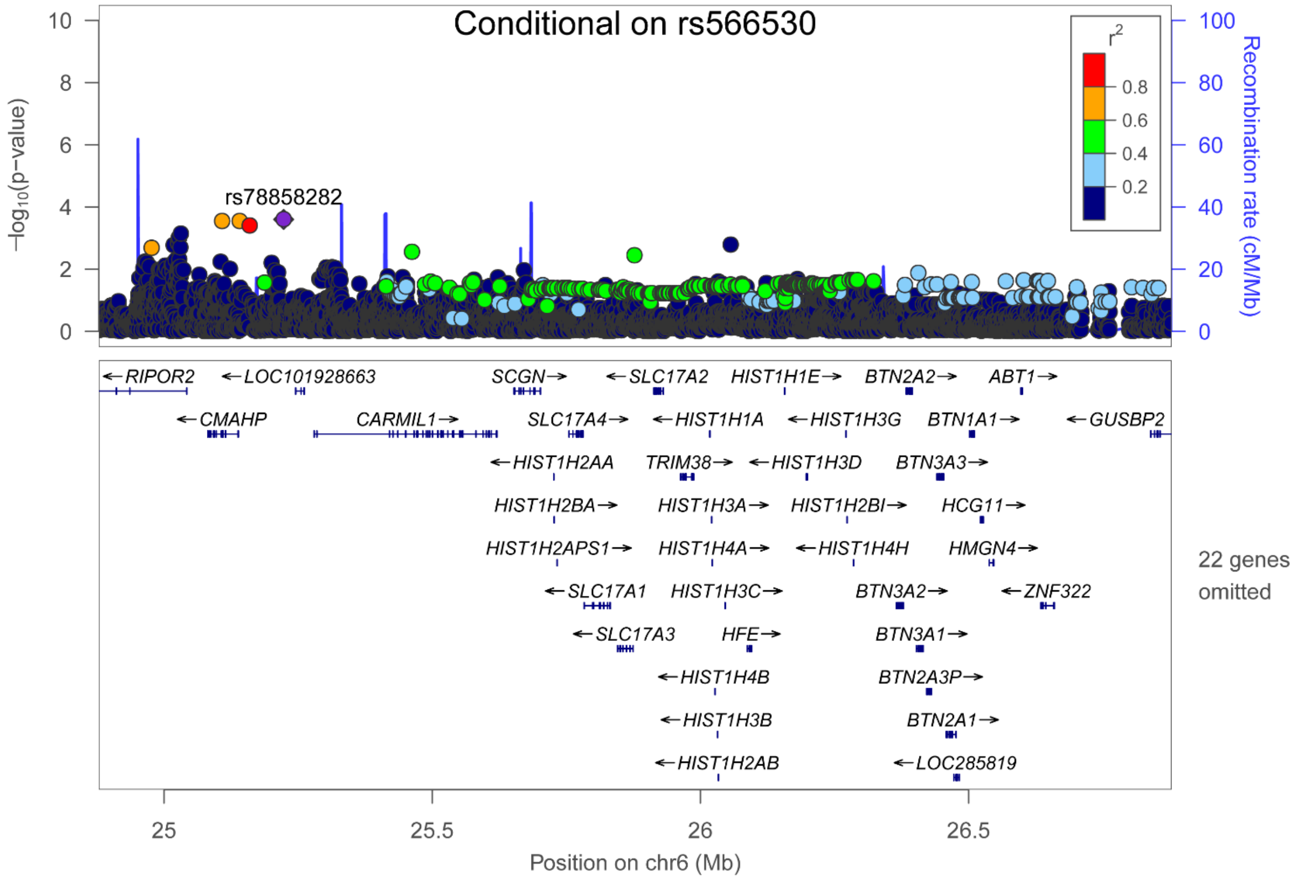

(**f**) Variants on chromosome 17 associated with SUA.

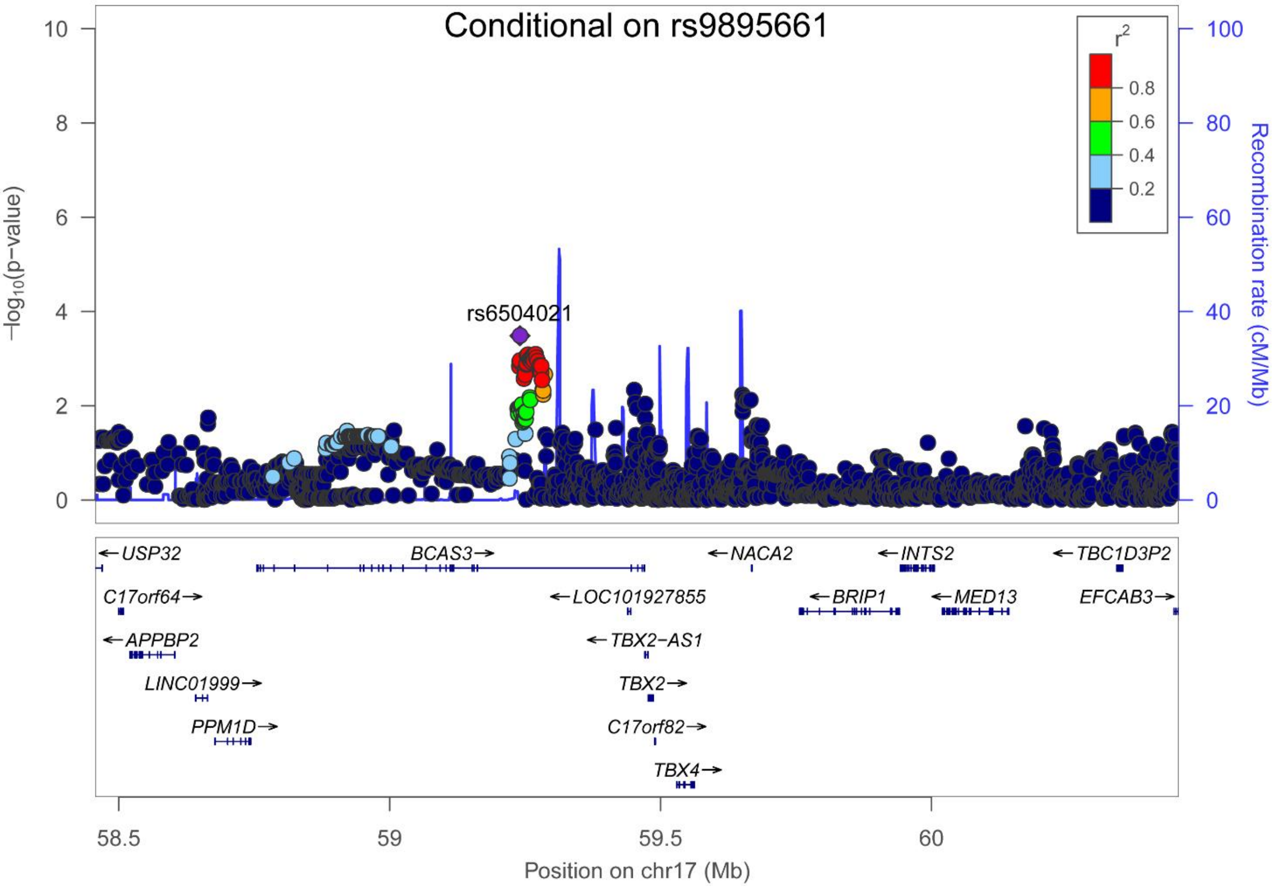

Fig. S5. Manhattan plot depicting genome-wide association analysis of serum uric acid (SUA) with simple covariates. Each dot represents a variant plotted as $-\log_{10}$(P-value) on the y axis against the corresponding variant position on the x axis. A genome-wide significance level is shown by the pink line. Age, sex, and the first 10 principal components derived from the genome-wide genotype data were adjusted.

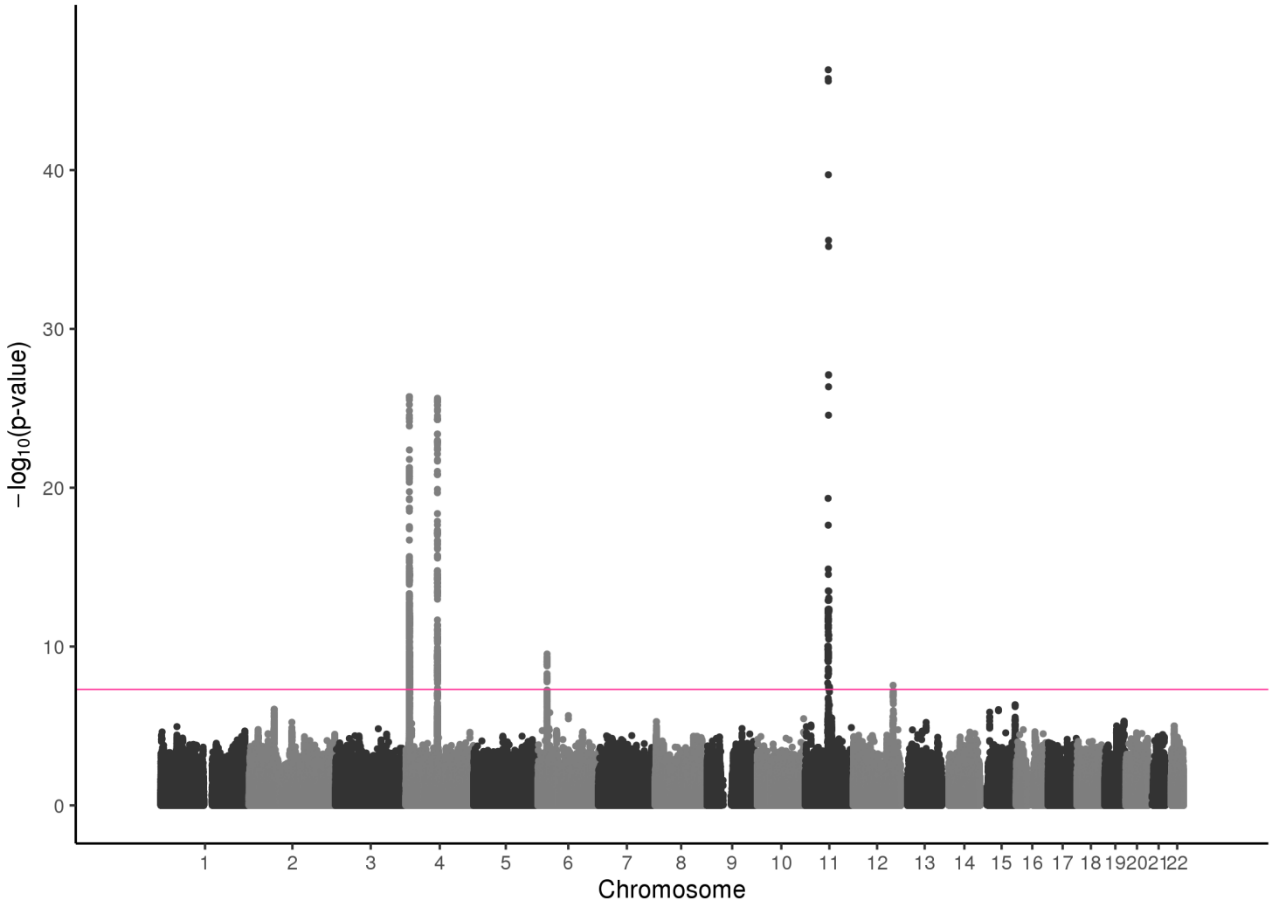

Fig. S6. Results of association analysis related to alcohol intake on chromosome 12. (**a**) Analysis of GWAS on SUA after adjusting for alcohol intake (Manhattan plot). (**b**) Variants on chromosome 12 associated with SUA after adjusting for alcohol intake. (**c**) Analysis of GWAS on alcohol intake (Manhattan plot). (**d**) Variants on chromosome 12 associated with alcohol intake.

(**a**) Analysis of GWAS on SUA after adjusting for alcohol intake (Manhattan plot).

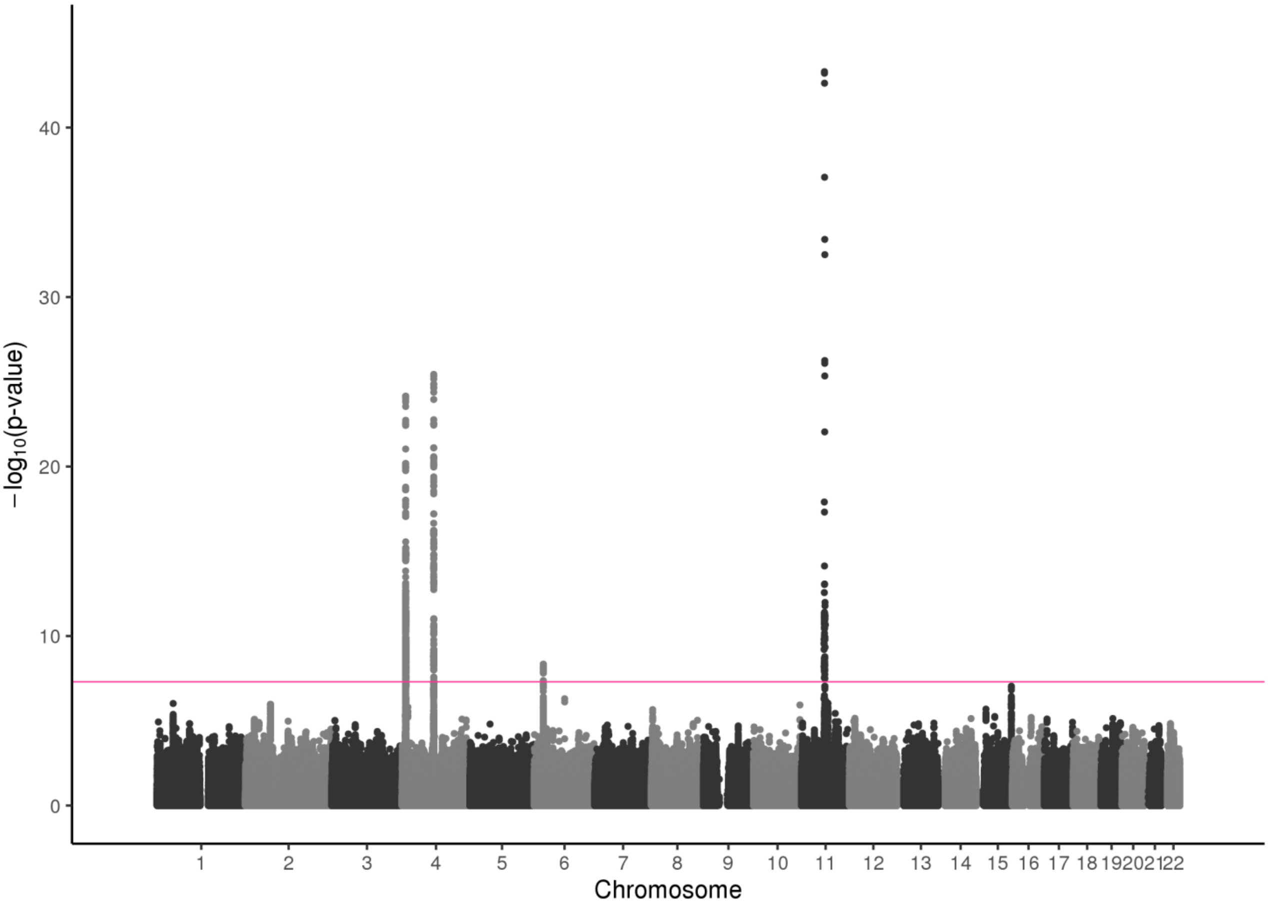

(**b**) Variants on chromosome 12 associated with SUA after adjusting for alcohol intake.

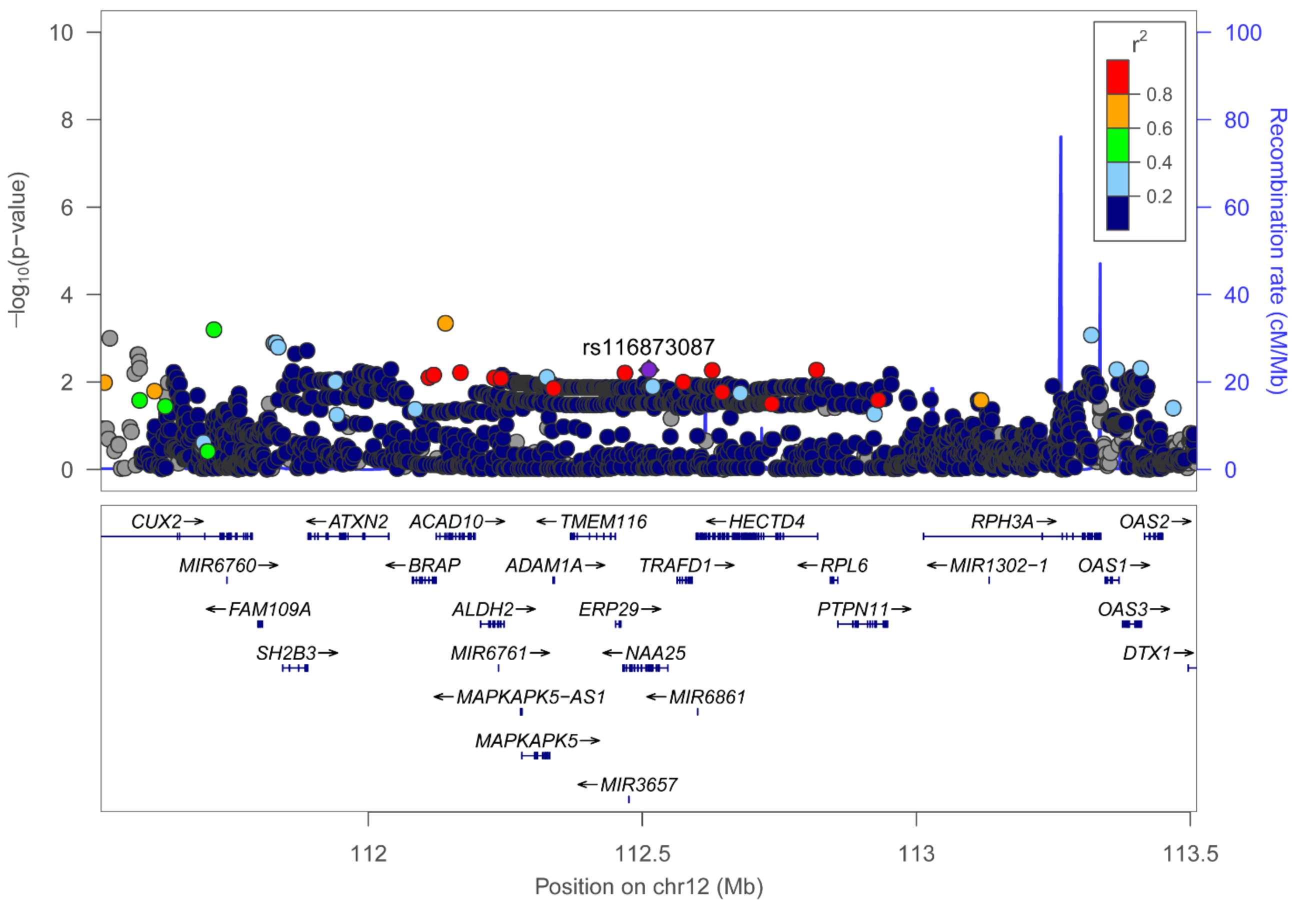

(**c**) Analysis of GWAS on alcohol intake (Manhattan plot).

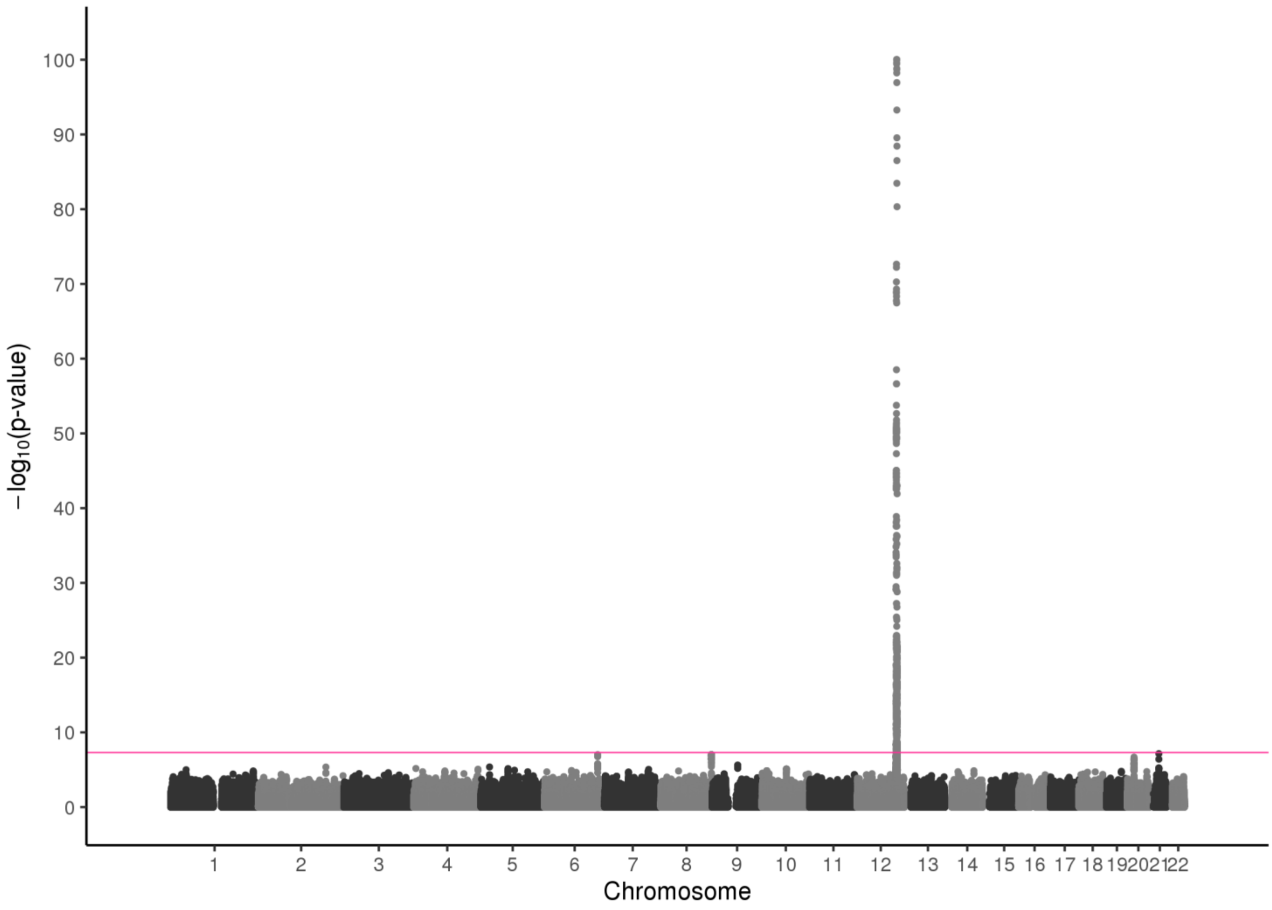

(**d**) Variants on chromosome 12 associated with alcohol intake.

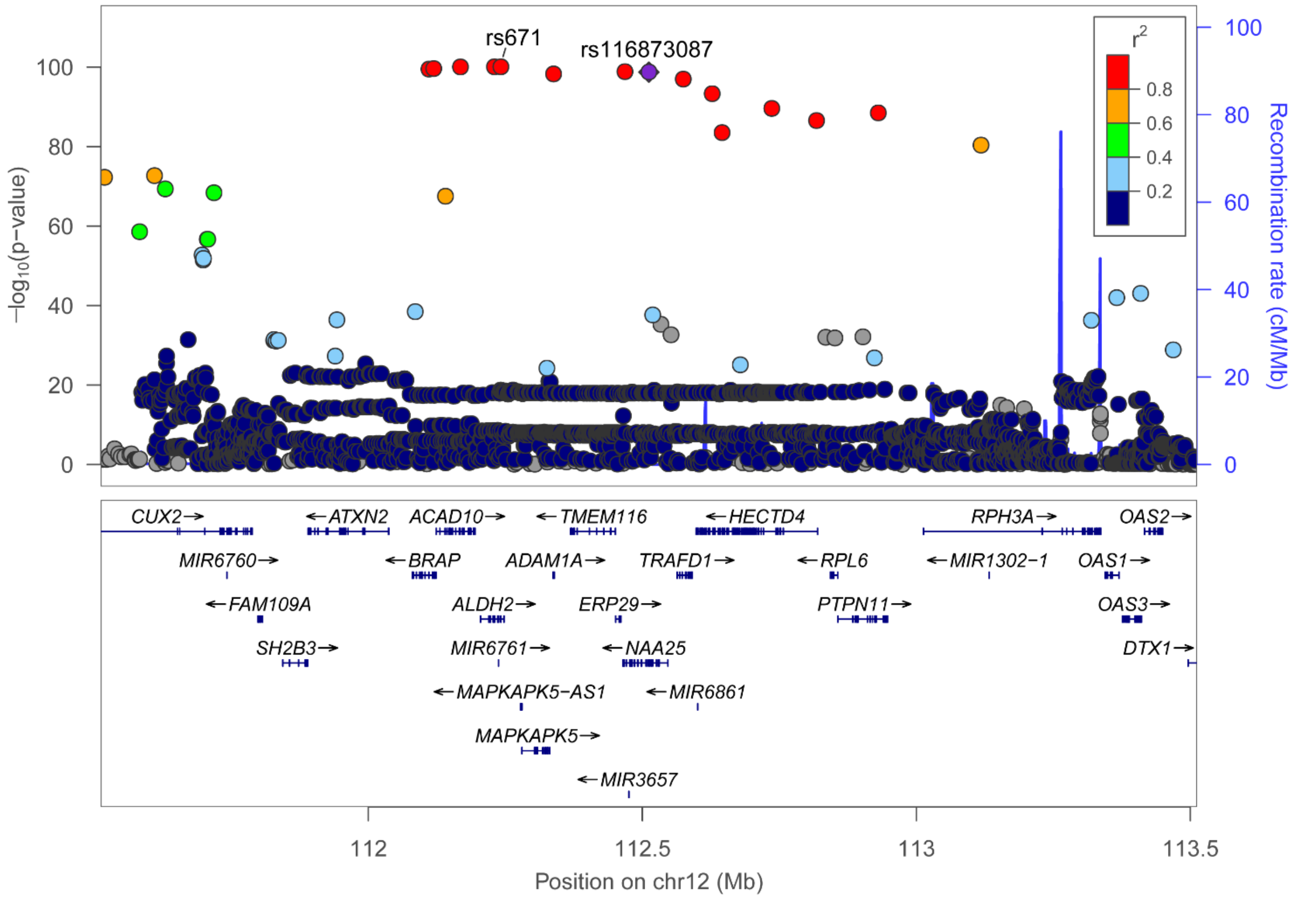

Fig. S7. Results of sex-stratified association analysis. Each dot represents a variant plotted as $-\log_{10}$(P-value) on the y axis against the corresponding variant position on the x axis for (**a**) male and (**b**) female participants. (**c**) Variants on chromosome 16 associated with SUA in female participants.

(**a**) Analysis of GWAS on SUA in male participants.

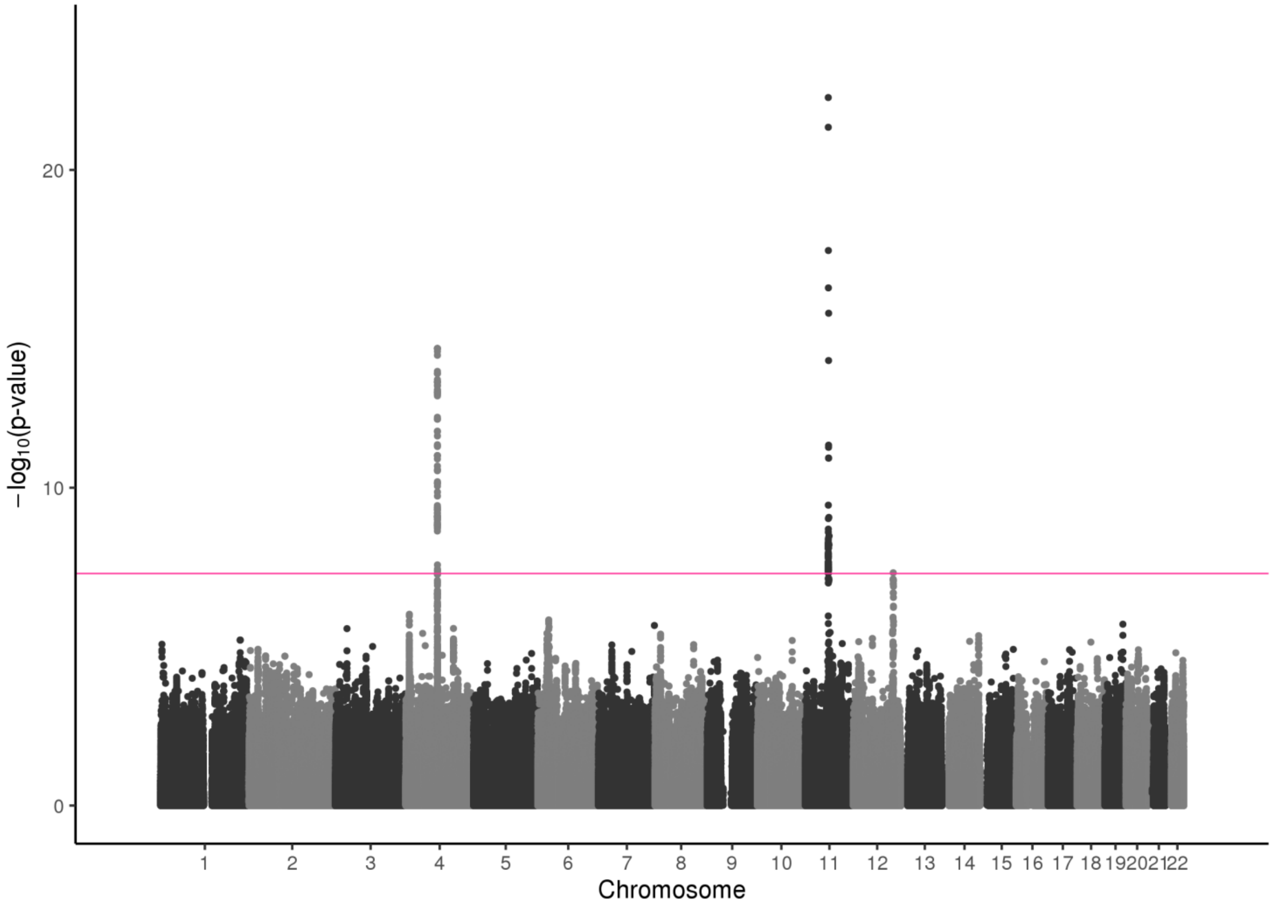

(**b**) Analysis of GWAS on SUA in female participants.

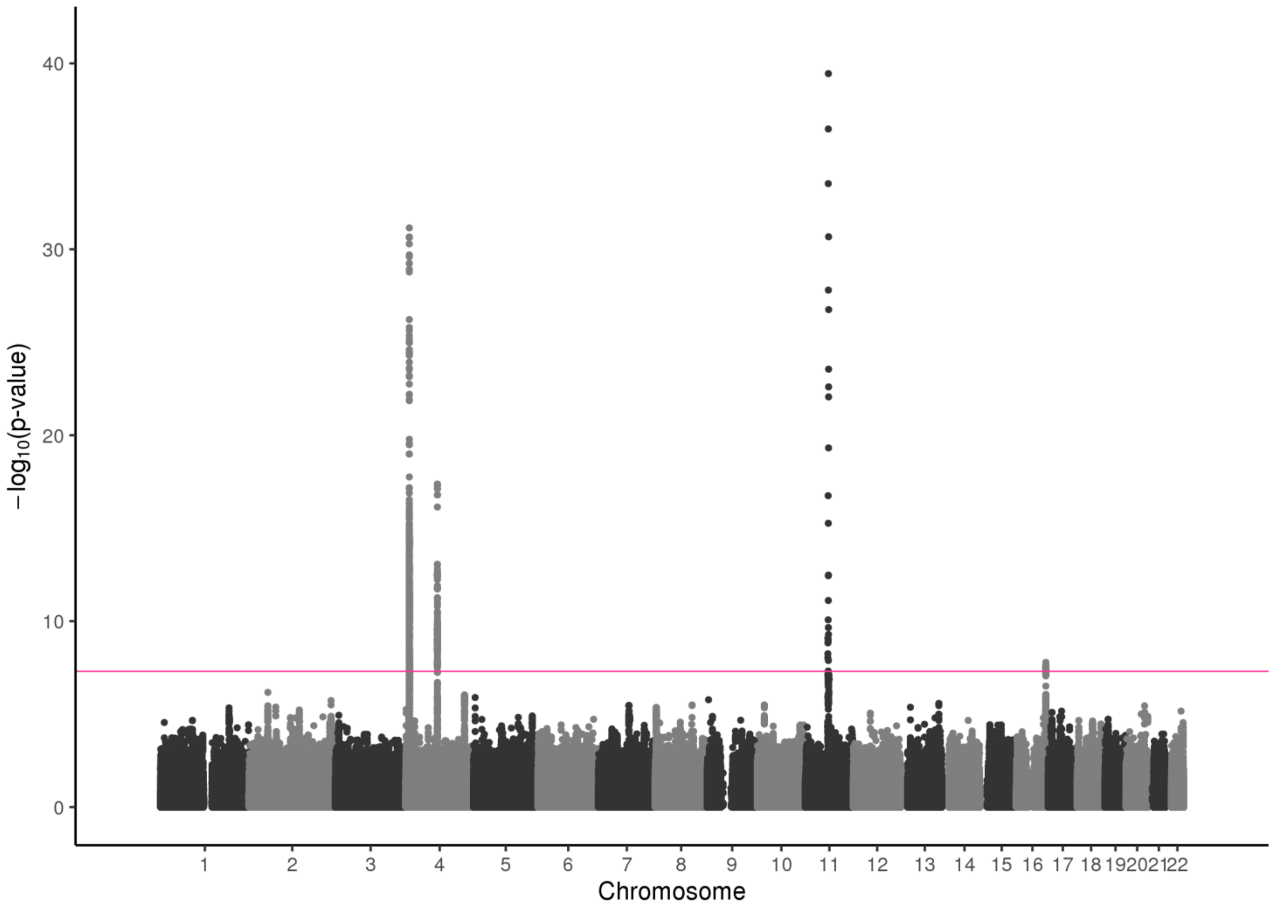

(**c**) Variants on chromosome 16 associated with SUA in female participants.

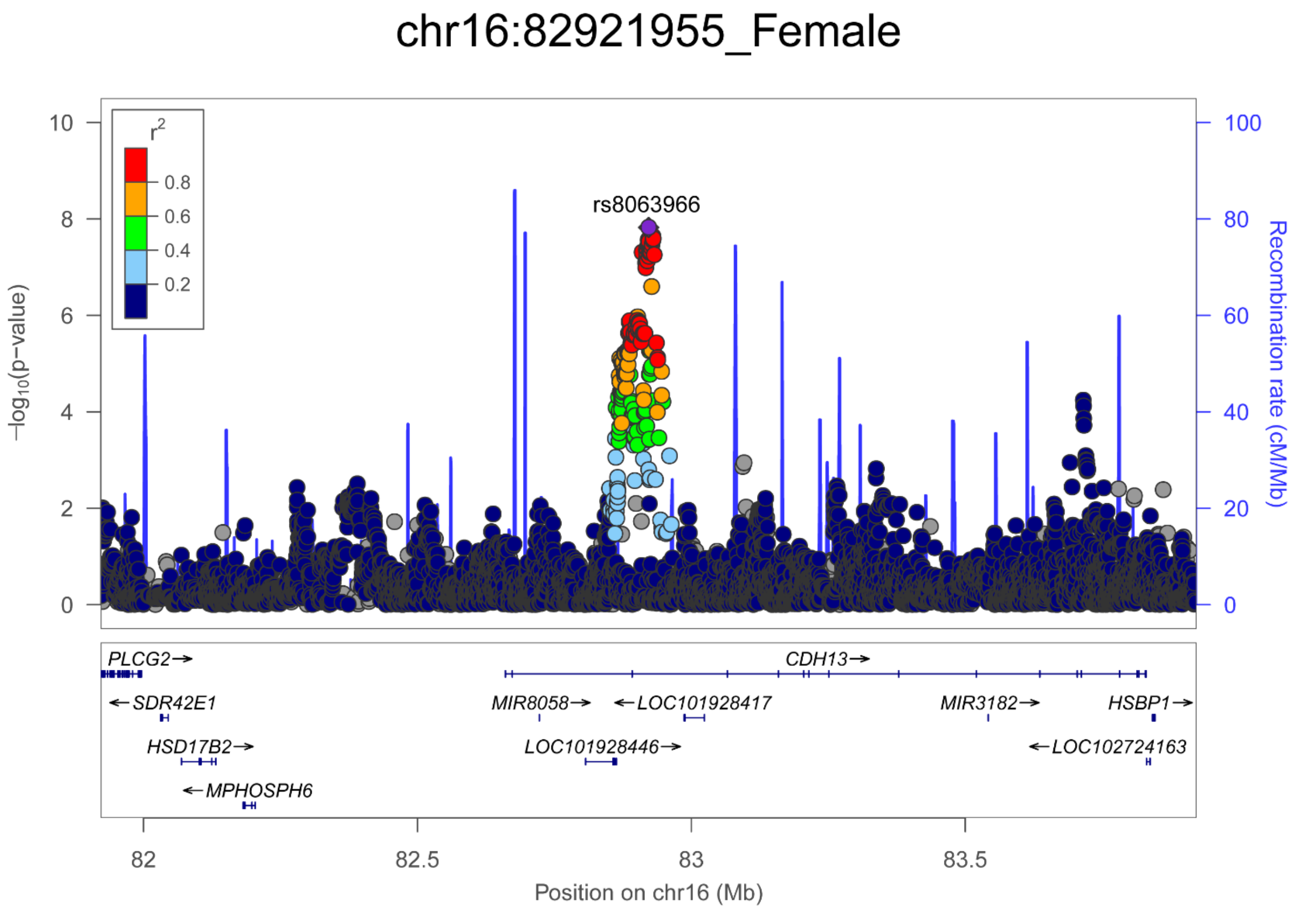

Supplement: Supplementary file 1 — Supplementary Tables and Figures. [file 41598_2020_66064_MOESM1_ESM.pdf]
